# Interplay of condensation and chromatin binding underlies BRD4 targeting

**DOI:** 10.1091/mbc.E24-01-0046

**Published:** 2024-05-21

**Authors:** Amy R. Strom, Jorine M. Eeftens, Yury Polyachenko, Claire J. Weaver, Hans-Frederick Watanabe, Dan Bracha, Natalia D. Orlovsky, Chanelle C. Jumper, William M. Jacobs, Clifford P. Brangwynne

**Affiliations:** a Department of Chemical and Biological Engineering, Princeton University, Princeton, NJ 08544; c Department of Chemistry, Princeton University, Princeton, NJ 08544; d Department of Molecular and Cellular Biology, Princeton University, Princeton, NJ 08544; h Omenn-Darling Bioengineering Institute, Princeton University, Princeton, NJ 08544; b Radboud Institute for Molecular Life Sciences, Radboud University, 6525 XZ Nijmegen, Netherlands; e Department of Biotechnology and Food Engineering, Technion, Haifa 3200, Israel; f Biological and Biomedical Sciences Program, Harvard University, Boston, MA 02115; g Nereid Therapeutics, Boston, MA; i Howard Hughes Medical Institute, Chevy Chase, MD 20815; Carnegie Mellon University

## Abstract

Nuclear compartments form via biomolecular phase separation, mediated through multivalent properties of biomolecules concentrated within condensates. Certain compartments are associated with specific chromatin regions, including transcriptional initiation condensates, which are composed of transcription factors and transcriptional machinery, and form at acetylated regions including enhancer and promoter loci. While protein self-interactions, especially within low-complexity and intrinsically disordered regions, are known to mediate condensation, the role of substrate-binding interactions in regulating the formation and function of biomolecular condensates is underexplored. Here, utilizing live-cell experiments in parallel with coarse-grained simulations, we investigate how chromatin interaction of the transcriptional activator BRD4 modulates its condensate formation. We find that both kinetic and thermodynamic properties of BRD4 condensation are affected by chromatin binding: nucleation rate is sensitive to BRD4–chromatin interactions, providing an explanation for the selective formation of BRD4 condensates at acetylated chromatin regions, and thermodynamically, multivalent acetylated chromatin sites provide a platform for BRD4 clustering below the concentration required for off-chromatin condensation. This provides a molecular and physical explanation of the relationship between nuclear condensates and epigenetically modified chromatin that results in their mutual spatiotemporal regulation, suggesting that epigenetic modulation is an important mechanism by which the cell targets transcriptional condensates to specific chromatin loci.

## Abstract (executive summary)

The role of substrate-binding interactions in regulating the formation and function of biomolecular condensates is under-explored.

Studying BET family transcription factor BRD4, the authors performed coarse-grained simulations and live cell experiments to elucidate the kinetic and thermodynamic properties of condensation that are affected by acetylated chromatin binding. They found that acetylated chromatin binding targets and enhances BRD4 condensation.

This provides a molecular and physical explanation of the relationship between nuclear condensates and epigenetically modified chromatin that results in their mutual spatiotemporal regulation, suggesting that epigenetic modulation is an important mechanism by which transcriptional condensates target specific chromatin loci.

## INTRODUCTION

Phase separation is a process by which molecules within living cells can be concentrated into biomolecular condensates, membraneless compartments that promote enzymatic activities at targeted sites (Zhang *et al.*, 2021; Saini and Mukherjee, 2023). Within the nucleus, multiple condensates exist that organize specialized functions within the complex nuclear environment, including ribosome production within nucleoli (Falahati *et al.*, 2016; Boisvert *et al.*, 2007), mRNA splicing within nuclear speckles (Xing *et al.*, 1993, 1995; Moen *et al.*, 2004; Brown *et al.*, 2008), as well as chromatin remodeling and transcriptional initiation within transcription factor condensates (Boija *et al.*, 2018; Sabari *et al.*, 2018; Ma *et al.*, 2021; Zhang *et al.*, 2021; Lyons *et al.*, 2023; Patil *et al.*, 2023). Formation of these nuclear condensates at specific genomic loci is essential for their function: nucleoli form at rDNA (Falahati and Wieschaus, 2017), nuclear speckles associate with highly transcribed genes (Xing *et al.*, 1993, 1995; Moen *et al.,* 2004; Brown *et al.,* 2008), and localized condensation of chromatin remodelers and transcription factors dictates gene expression (Sabari *et al.*, 2018; Patil *et al.*, 2023). Still, both experimental and simulation studies of the biophysical determinants of condensation have, to date, focused primarily on describing interactions that drive condensate formation, leaving the mechanisms underlying their genomic targeting relatively unstudied. Transcription factors and coactivators often contain dual modalities: both self-interaction motifs to promote condensation, and chromatin-binding motifs to interact with certain genomic sequences or epigenetic marks, though the biophysical interplay of these multiple types of interactions and their potential to drive localized condensation have not been fully explored.

Condensation is driven by thermodynamic demixing of biomolecules due to transient multivalent interactions, which are commonly provided by intrinsically disordered regions (IDR) of proteins, though multivalent interactions can also arise from folded protein regions or from binding to substrates including RNA or chromatin (Villegas, Heidenreich, and Levy, 2022). When the concentration of a phase separation–prone protein is above a threshold called the saturation concentration, it becomes energetically favorable for the solution to phase separate, forming a continuous dilute and a discrete dense phase of droplets (Banani *et al.*, 2017; Shin and Brangwynne, 2017; Posey *et al*., 2018). Phase separation–prone proteins including transcription factors and coactivators often incorporate both homotypic (self-self) and heterotypic (self-nonself) interactions, both of which may modify the condensation properties of the phase separating system (Shin, 2022; Farag *et al.*, 2023). Interactions that modify the conditions under which phase separation occurs are characterized as affecting equilibrium thermodynamic properties, which in inanimate systems are often associated with temperature (Sariban and Binder, 1988) and in living systems are often associated with composition, concentration, and valence of the phase separation–prone molecules (Hyman *et al*., 2014; Bracha *et al.*, 2019).

Targeted condensation can occur through seeded nucleation on a substrate, a process that is driven by kinetic, or rate-dependent, properties of the phase separating system. Classical nucleation theory describes the initial formation of a condensed phase from a homogenous solution, which occurs when freely diffusing monomers begin to gather into dynamic nanoscale clusters (Kashchiev, 2000). These clusters continually form and dissolve, until one exceeds the critical radius, at which point it is more energetically favorable to grow than shrink. Many features of the nucleation of biomolecular condensates are in agreement with classical nucleation theory, including the strong dependence of nucleation rate on the degree of supersaturation (Wei *et al.*, 2020; Wiegand and Hyman, 2020; Shimobayashi *et al.*, 2021). Targeted nucleation can be achieved by lowering the energetic barrier of nucleation through binding monomers to a substrate or “seed,” which reduces the critical radius required for continued growth, in a process referred to as heterogeneous nucleation (Kashchiev, 2000; Chayen *et al*., 2006). Simulations have demonstrated that seeded nucleation results in faster and spatially localized condensate formation, and may be an important mechanism through which functional endogenous condensates are targeted to form at specific genomic loci (Shin *et al.*, 2018; Wei *et al.*, 2020; Shimobayashi *et al.*, 2021), though this has not yet been experimentally demonstrated.

Transcriptional initiation condensates are an ideal model to study the kinetic and thermodynamic contributions of chromatin substrate binding to functional biomolecular phase separation, as the ability of transcriptional activators to both condense and bind chromatin is well established (Plys and Kingston, 2018; Sabari *et al.*, 2018; Ma *et al.*, 2021; Wagh *et al*., 2021), and their targeting to specific genomic loci is functionally relevant in dictating the cell’s transcriptional profile. The BET family protein BRD4 is a well-studied transcriptional activator that localizes to acetylated chromatin sites (Dey *et al.*, 2003; Wu *et al.*, 2018), recruits pTEF-b (Itzen *et al.*, 2014) and initiates transcription of key genes involved in signal response, immunity, and oncogenesis (Wang *et al.*, 2021). All Bromodomain and Extra-terminal (BET) family proteins contain Extra-terminal (ET) domains (BRD2, BRD3, BRD4, and BRDT) bind to lysine-acetylated histones through two conserved N-terminal bromodomains, and recruit additional cofactors through the shared ET domain. However, only BRD4 can directly engage pTEF-b through a C-terminal motif (CTM) in its extended, unstructured C-terminus. Moreover, while the expression of BRD2, BRD3, and BRDT are tissue-specific, BRD4 expression is ubiquitous (Wang *et al.* 2021). BRD4’s C-terminal ∼1000 amino acid IDR has been characterized as a driver of self-interaction and condensate formation in living cells (Sabari *et al.*, 2018; Shin *et al.*, 2018; Wang *et al.*, 2019; Han *et al.*, 2020), which has been implicated in its enhanced ability to drive high levels of transcription.

For BRD4 specifically, condensate-forming capabilities of the C-terminal IDR are thought to occur through self-interactions, or in combination with MED1 (Sabari *et al.*, 2018), which contributes to the thermodynamic driving force for phase separation. Localization to specific chromatin regions is mediated through the two N-terminal bromodomains, which bind acetylated histones and thereby serve as reader domains to mediate genomic interaction. The inherent multivalency of acetylated regions on the genome may not only provide targeting sites that can enhance kinetics, but could also contribute thermodynamically to BRD4 phase separation (Sabari *et al.*, 2018; Morin *et al.*, 2022). Indeed, substrates with repetitive binding sites may enhance the valence of the condensing system and allow formation of condensates at lower concentration. This is seen with other nucleic acid–binding proteins like FUS, which is capable of condensation without RNA binding, while addition of RNA reduces the critical concentration (Maharana *et al.*, 2018; Niaki *et al.*, 2020). However, it remains to be elucidated how the interplay of kinetic and thermodynamic properties of transcriptional initiation condensates facilitate their formation at targeted chromatin loci.

Here we use coarse-grained simulation and quantitative experiments in living cells to decode the biophysical rules underlying how interactions between chromatin and proteinaceous condensates of BRD4 impact condensate formation and targeting. We show that binding to acetylated chromatin regions allows BRD4 condensation at lower valence than is required for off-chromatin, and that these acetylated regions act as heterogeneous nucleation seeds for BRD4 condensates. We propose that the cell regulates both kinetic and thermodynamic properties of condensation to control the spatiotemporal localization of transcriptional condensates for their nuclear function.

### Disruption of BRD4 chromatin binding reduces number and volume of condensates

**FIGURE 1:**
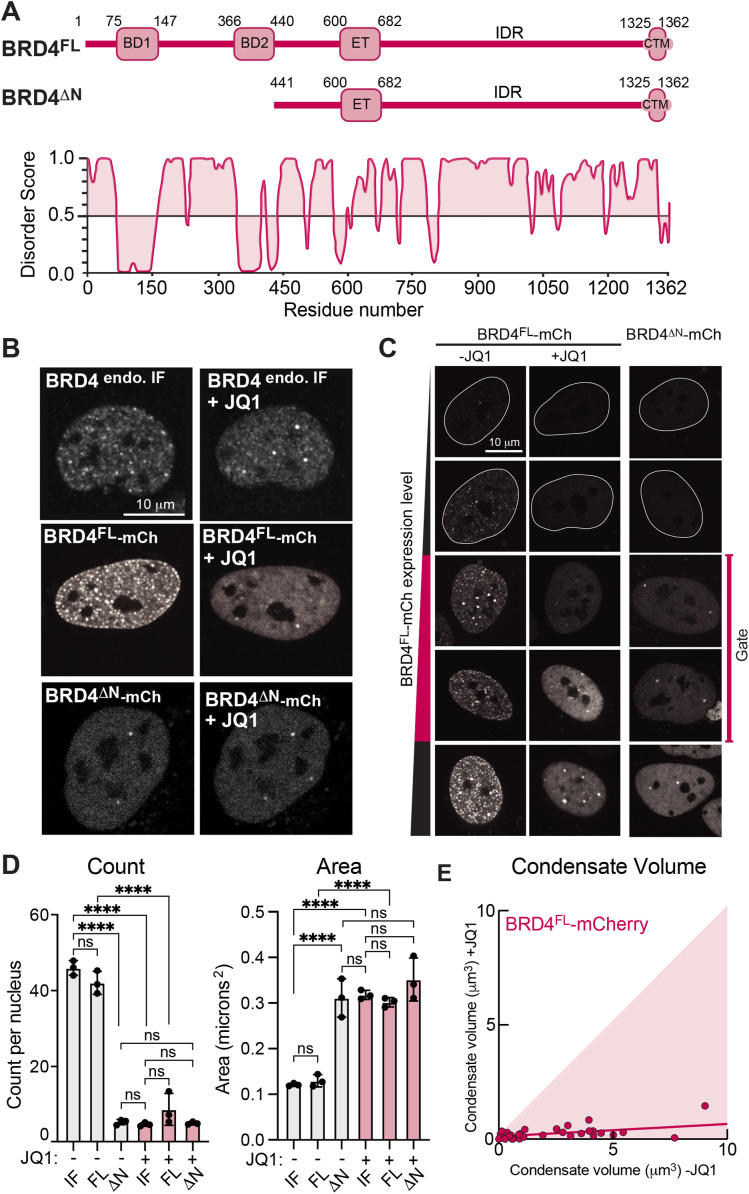
Chromatin binding influences BRD4 condensate distribution in living cells. (A) Schematic of BRD4 full-length (FL) and deltaN-terminus (∆N) constructs, as well as PONDR predicted disorder score. (B) From top: Immunofluorescence of endogenous BRD4 protein in cultured human U2OS cells without and with addition of 1 µM JQ1. Exogenous live expression of BRD4^FL^-mCherry and BRD4^∆N^-mCherry in the same cell before (−JQ1) and after (+JQ1) addition of 1 µM JQ1. (C) Panel of images of U2OS cells with increasing expression level of BRD4^FL^-mCherry (same cell −/+ JQ1) or BRD4^∆N^-mCherry at similar expression levels. (D) Quantification of number and size of BRD4 condensates from immunofluorescence of endogenous, or live expression of BRD4^FL^-mCherry or BRD4^∆N^-mCherry, −/+ JQ1. Points represent averages of three biological replicates of 25 cells each within the expression level gate defined in 1C, error bars SEM. Statistical test one-way ANOVA, ****p < 0.0001. (E) Estimated condensate volume measured in the same set of cells expressing BRD4^FL^-mCherry before (*x*-axis) and after (*y*-axis) disruption of chromatin binding through addition of 1 µM JQ1. If condensate volume is not affected, points should lie on the diagonal, indicated by shading.

The protein structure of BRD4 includes two-folded N-terminal bromodomains (BD1 aa 75-247; BD2 aa 360-440) which bind to acetylated chromatin (Ji *et al.*, 2015), and a CTM (aa 1047-1362) that interacts with pTEF-b, as well as the large C-terminal IDR which coordinates self-interaction and is known to contribute to BRD4 phase separation (Sabari *et al.*, 2018; Shin *et al.*, 2018) (Figure 1A). By immunofluorescence, and in agreement with previous reports (Boija *et al.*, 2018; Sabari *et al*., 2018), BRD4 forms punctate nuclear structures under endogenous expression levels in U2OS cells, which are recapitulated with expression of fluorescently-tagged BRD4^FL^-mCherry in living nuclei (Figure 1B). To examine the role of chromatin binding in BRD4 condensation and targeting, we utilized the small-molecule BET inhibitor (BETi) JQ1, which can specifically disrupt interaction of bromodomains with acetylated chromatin (Filippakopoulos *et al.*, 2010; Jiang *et al.*, 2020), and leads to eviction of BRD4 from chromatin sites (Donato *et al.*, 2017). Upon treatment of cells with 1 μM JQ1 for 90 min, we find that BRD4 becomes dispersed throughout the nucleoplasm, with few large foci remaining per nucleus in both the endogenous immunofluorescence and exogenous expression cases (Figure 1B). This altered nuclear localization is phenocopied by expression of the truncated construct BRD4^∆N^-mCherry (aa 441-1362) in which both bromodomains are removed (Figure 1, A and B). A panel of cells arranged by expression level of BRD4^FL^-mCherry shows that cells with higher expression have more puncta (Figure 1C). Nonetheless, across expression levels, the number of puncta in individual cells after JQ1 treatment is consistently lower than before treatment, visually resembling cells expressing BRD4^∆N^-mCherry at similar expression levels (Figure 1C). To avoid BRD4 expression–dependent effects in quantification, we used the number and size of endogenous BRD4 puncta by immunofluorescence to create lower and upper gates on the relevant expression levels of BRD4^FL^-mCherry (Figure 1C), such that within this range, the quantification of the number and size of live expression BRD4^FL^-mCherry condensates is indistinguishable from quantification of the endogenous protein by immunofluorescence. This analysis further confirms that disruption of chromatin binding with JQ1 results in fewer, larger puncta that match those formed by BRD4^∆N^-mCherry (Figure 1D).

These findings demonstrate that the number, size, and positioning of BRD4 condensates in the nucleus is altered by chromatin-binding capabilities. A simple explanation is that disruption of chromatin binding releases condensates from defined loci, allowing them to coalesce into fewer, larger droplets (Feric and Brangwynne, 2013; Caragine, Haley, and Zidovska, 2018; Lee *et al.*, 2023). If the alteration of condensate number and size is purely due to kinetic effects, we would expect the total volume of condensed material in each cell to remain consistent before and after addition of JQ1, such that a set volume of many small droplets reorganizes into fewer, larger ones. To test this, we measured the cross-sectional area of BRD4^FL^-mCherry condensates in the same nuclei before and after JQ1 treatment, then estimated the volume of condensates in each nucleus (see *Methods*). Surprisingly, we find that the volume of BRD4^FL^-mCherry condensates is substantially decreased upon JQ1 treatment (Figure 1E), suggesting that disruption of BRD4’s chromatin binding does not simply lead to coalescence of small droplets, but also impacts the thermodynamics of BRD4 condensation. Together, these data illustrate a complex role for chromatin binding in regulating both kinetic and thermodynamic properties of BRD4 condensation that warrants further quantitative investigation.

### Chromatin binding thermodynamically enhances BRD4 condensation

We next sought an experimental system that would be capable of quantitatively investigating the concentration-dependent thermodynamics of BRD4 phase separation, while also having a well-defined trigger for initiating condensate formation to study kinetics. The Corelet system is a two-component platform capable of triggering phase separation in living cells through light-induced oligomerization of an sspB-tagged phase separation–prone protein domain via interaction with 24-mer multivalent iLID-GFP-Ferritin “Cores” (Supplemental Figure S1A) (Bracha *et al.*, 2019). The Corelet system can be used to build a phase diagram to map the thermodynamic properties of BRD4 phase separation by quantitatively determining the Core concentration and BRD4-to-Core valence required for BRD4^FL^ condensate formation in untreated (Control) or JQ1-treated cells (with disrupted chromatin binding).

**FIGURE 2:**
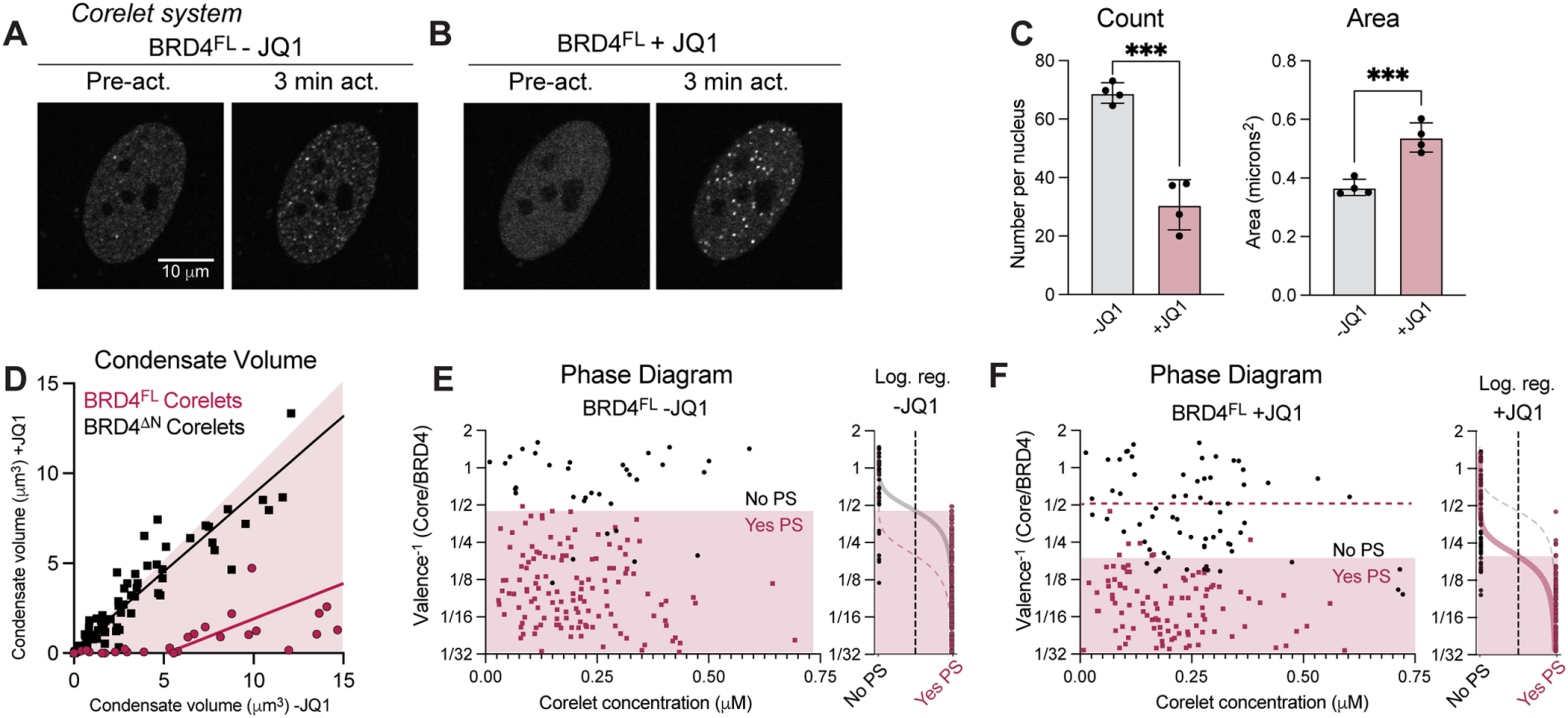
Thermodynamic effects of BRD4 chromatin binding are measured the Corelet synthetic oligomerization system. Representative images of BRD4^FL^ Corelets before and after light activation of a nucleus without (A) or with (B) 1µM JQ1. (C) Quantification of the number and size of BRD4^FL^ Corelet condensates per nucleus induced with light activation −/+ JQ1 in the same set of nuclei. Error bars represent SEM across four trials of 25 cells each, Student’s *t* test ***p = 0.0002 in Count, *** *p* = 0.001 in Area. (D) Condensate volume comparison in −/+JQ1 conditions in the same set of cells with BRD4^FL^ (pink) or BRD4^∆N^ (black) Corelets. BRD4^∆N^ Corelet condensate volume is unaffected by the addition of JQ1, as can be seen from points largely along the diagonal (shaded pink), while BRD4^FL^ Corelet condensate volume is much lower after addition of JQ1. Lines are linear fit. (E, F) Phase diagram and logistic regression of BRD4^FL^ measured with the Corelet system in the absence (E) and presence (F) of JQ1. Phase diagrams are constructed from the same cells expressing BRD4FL −/+ JQ1 demonstrating the shifted valence between −JQ1 (gray) and +JQ1 (pink).

We expressed BRD4^FL^ Corelet components in U2OS cells and initiated their oligomerization with blue light (Figure 2A). Before light activation, live expression of BRD4^FL^-mCherry-sspB exhibits a few puncta preactivation, similar to the native condensates visualized by immunofluorescence (Figure 2A, left). Then, upon light-activated oligomerization, de novo BRD4^FL^ condensates form (Figure 2A, right). Because oligomerization in the Corelet system is reversed in the absence of blue light, we can compare the number and size of condensates in the same set of BRD4^FL^-mCh-sspB–expressing cells before and after addition of JQ1 (1 μM, 90 min), to determine whether the concentration and valence required for condensation are altered upon loss of chromatin binding. In the same cell with JQ1 treatment, light activation again leads to BRD4^FL^ Corelet condensate formation (Figure 2B), though the condensates are fewer and larger (Figure 2C), mimicking the changes observed upon disruption of chromatin binding in the endogenous and exogenous expression systems introduced earlier. JQ1 treatment of BRD4^∆N^ Corelet condensates has no effect on puncta number or size (Figure 2D; Supplemental Figure S1, B–E), confirming that JQ1 specifically disrupts chromatin binding, without affecting IDR-mediated condensate formation. The number of Corelet puncta per nucleus on a cell-by-cell basis before and after addition of JQ1 makes clear the drastic reduction in BRD4^FL^-mCh-sspB condensate number at most expression levels (Supplemental Figure S1E). Interestingly, cells with very high Corelet condensate count are less severely affected, likely because oligomerization through the Ferritin Core platform can substitute for oligomerization through chromatin binding at very high expression levels, making these cells less responsive to JQ1.

Calibrated fluorescence microscopy of cells expressing BRD4^FL^ Corelets can be used to construct an oligomerization- and concentration-dependent phase diagram, wherein proteins that form condensates at lower BRD4-to-Core valence have a stronger phase separation tendency (plotted as inverse valence, Core/BRD4). Compared with previously published examples of Corelet phase diagrams (Bracha *et al.*, 2019; Sanders *et al.*, 2020), extremely low Core concentration is sufficient to initiate BRD4^FL^ condensate formation, and we measure a critical valence of two BRD4^FL^-mCh-sspB bound per core required for condensate formation (shaded area, Figure 2E). This lack of a left-hand boundary at low Core concentration highlights that the nuclear interior is poised for BRD4 phase separation with minimal additional oligomerization and may reflect the presence of other interaction partners in the nucleus like MED1 (Sabari *et al.*, 2018). The presence of BRD4 interactors like MED1 in the nucleus could contribute to the preexisting puncta seen before light activation and make the saturation concentration appear lower than it would be for BRD4 alone (Supplemental Figure S1, F and G). When the valence distinction between phase-separating (Yes PS) and non–phase-separating cells (No PS) is measured in the same nuclei after disruption of chromatin binding with JQ1, it is shifted to five BRD4^FL^-mCh-sspB bound per core required for condensate formation (Figure 2F). This shift in required valence is readily apparent when the phase diagram data are quantified as a logistic regression (Figure 2, E and F). Together, these data indicate that BRD4 is capable of condensate formation in the Corelet system both with and without chromatin binding, but that chromatin binding provides a thermodynamic enhancement of BRD4’s phase separation tendency.

### Coarse-grained simulations characterize valence dependence of BRD4 condensation

**FIGURE 3:**
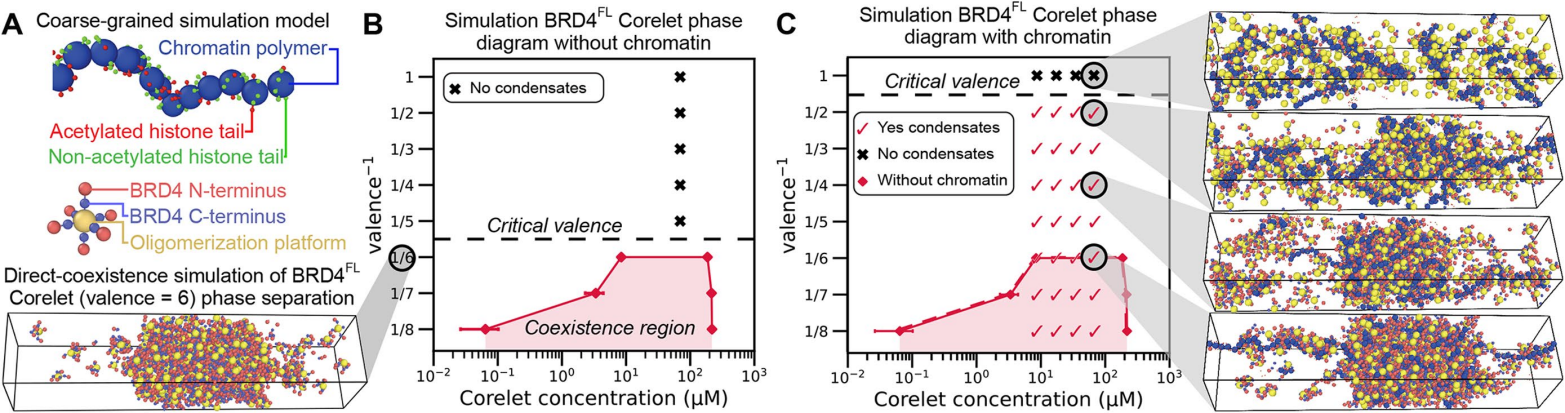
A coarse-grained model for simulating BRD4 Corelet condensation. (A) Coarse-grained simulations contain representations of the chromatin polymer (blue) and Corelet oligomerization platform (gold) with attached BRD4 molecules, each composed of two spheres representing the N-terminus (capable of interacting with chromatin) and C-terminus (capable of self-interaction). (B) The valence-dependent phase diagram in the absence of chromatin, obtained via direct-coexistence simulation (representative snapshot on left), shows a critical valence^-1^ of 1/6. **C.** A phase diagram showing the presence of chromatin-Corelet condensates in simulations with strong chromatin binding (40% acetylated histone tails) predicts an apparent critical valence^-1^ of 1/2. Representative snapshots are shown (on right) for the indicated simulation conditions. Related simulation data at lower acetylation levels are provided in Supplemental Figure S2F.

To better understand the thermodynamic and kinetic mechanisms by which chromatin binding affects BRD4 phase separation, we next created a coarse-grained simulation of BRD4 condensation. In this system (Figure 3A), the chromatin polymer is represented by a chain of nucleosome monomers, each with eight histone tails that can either be in an acetylated state, enabling attractive interactions with BRD4 (red), or in a noninteracting state (green). The ∼1400 amino acid BRD4 protein is represented as two soft spheres (Louis *et al.*, 2000) connected by a FENE potential, one representing the acetylated histone tail–interacting N-terminus (referred to as “chromatin-interacting” in what follows) and the other representing the disordered self-interacting C-terminus. Model BRD4 Corelets of variable valence are created by tethering BRD4 molecules to a central oligomerization platform via their C-termini, mimicking the geometry of these constructs in the Corelet system.

Simulations of model BRD4 Corelets can be used to create phase diagrams that parallel the experimental Corelet phase diagrams from Figure 2, both in the presence and absence of interactions with acetylated chromatin. Direct-coexistence simulations (Dignon *et al.*, 2018) of model Corelets indicate that the critical valence for phase separation in the absence of chromatin is between 5 and 6 BRD4 molecules per “Core” oligomerization platform (Figure 3B), which is in close agreement with the experimental measurement of critical valence from Figure 2F. We note that a key difference between the simulation and experimental phase diagrams is that all simulations are performed with a monodisperse valence distribution, whereas Corelets in experiments likely have a broader valence distribution. Broadening the valence distribution tends to reduce the apparent saturation concentration for phase separation, since higher-valent species tend to phase-separate at lower concentrations (Bianchi *et al.*, 2006). When chromatin with 40% acetylated histone tails is added, which is a reasonable approximation for highly acetylated active regions (Crouch *et al.*, 2007; Kang *et al.*, 2020), this results in the formation of chromatin-enriched condensates at low valence, in conditions where BRD4 Corelets are unable to condense on their own (Figure 3C). We also created phase diagrams with chromatin acetylated at 20 and 30% of histone tails, and find that the critical Corelet valence for the formation of finite-size condensates is indeed dependent on the percentage of histone tails that are acetylated (Supplemental Figure S2F). These simulations indicate that the critical valence is two BRD4 molecules per oligomerization platform in the presence of 40% acetylated chromatin, due to a combination of chromatin–BRD4 and BRD4–BRD4 attractive interactions (Broedersz *et al.*, 2014; Erdel and Rippe, 2018). This is again in striking agreement with experimental findings (Figure 2E).

Importantly, our simulations provide an explanation for the critical valence difference between the −/+ JQ1 cases in experiments, since the elimination of chromatin–BRD4 interactions reverts the phase behavior to that of the Corelet system without chromatin. Moreover, the simulations suggest that puncta visualized at very low valence in the −JQ1 experimental case do not represent conventional phase separation, but rather subsaturation clusters stabilized by regions of highly acetylated chromatin, because the highly acetylated chromatin is a necessary component of this structure. In our simulations, these clusters do not grow to be much larger than the globule of compacted chromatin. BRD4 clustering in this scenario does not correspond to a unique coexistence pressure, which would determine the saturation concentration of Corelets in conventional phase separation. Consistent with this interpretation, condensates with valence^–1^ between 1/2 and 1/5 are limited in volume (Supplemental Figure S2, A and B) and entirely dissolve upon disruption of chromatin binding (Supplemental Figure S2C). Quantification of the fraction of Corelets in the dense (liquid) phase in simulated condensates (Supplemental Figure S2D) is consistent with measured volume of condensates in Corelet experiments (Supplemental Figure S2C) and demonstrates that below the critical valence^–1^ (1/6, dotted line in Supplemental Figure S2D), we do not observe a consistent dense phase fraction. See representative example images and movies of Corelet activation of BRD4^FL^-mCh-sspB in −/+ JQ1 conditions in Supplemental Figure S2E and Supplemental Movie S1. Together, these data indicate that chromatin is an integral part of BRD4 condensates that form at low BRD4-to-Core ratios and at low Corelet concentrations, suggesting that chromatin interactions can affect the thermodynamics of BRD4 condensate formation.

**Figure:** Movie S1

### Chromatin acts as a heterogeneous seed for BRD4 condensate nucleation in cells

**FIGURE 4:**
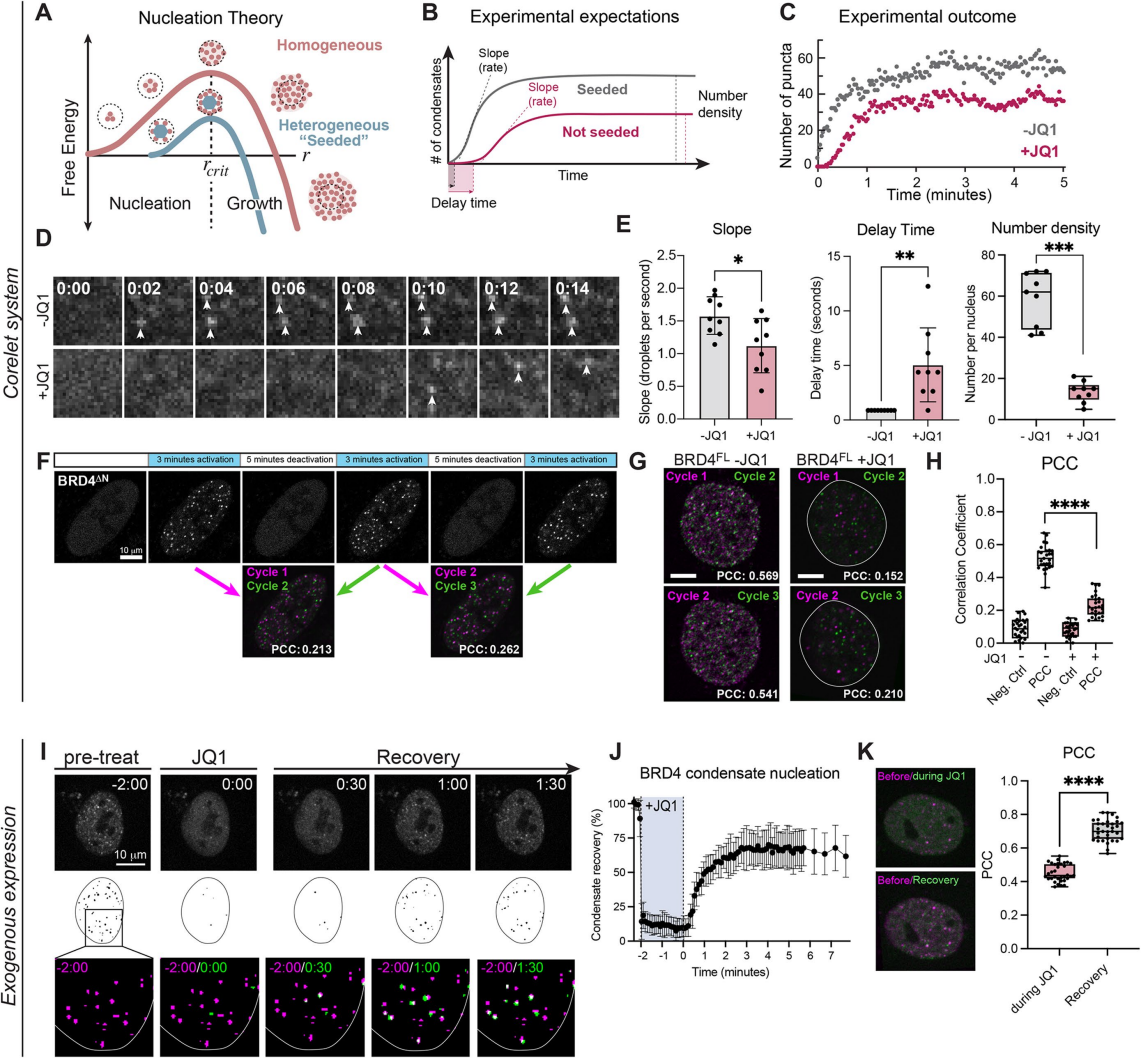
BRD4 condensate nucleation is seeded on chromatin. (A) Schematic of two modes of condensate nucleation: substrate-seeded (heterogeneous) and not seeded (homogeneous). Without seeding, the free energy of protein clustering increases until the cluster reaches its critical radius (*r_crit_*), at which point it becomes energetically favorable to grow. A seed can lower the energetic barrier to reach *r_crit_*. (B) Seeded nucleation is expected to have a shorter delay time before droplet formation, and increased nucleation rate (slope) compared with nonseeded nucleation. (C) Quantification of the number of condensates nucleated over time in the same cell before and after JQ1 treatment demonstrates the expected changes in delay time and slope. (D) Representative images of a 4 by 4 micron square nuclear area as light-induced BRD4^FL^-mCh-sspB Corelet condensates nucleate rapidly in untreated conditions (2 s, top), but are delayed in the same cell after JQ1 treatment (10 s, bottom). (E) Quantification of the nucleation rate (slope), delay time, and number density for 9 cells of similar expression level before and after JQ1 treatment. (F) Repeated activation-deactivation cycles of BRD4^∆N^ and overlay of condensate positions in subsequent cycles shows whether nucleation occurs repeatedly in the same nuclear locations. (G) Overlay of condensate positions in subsequent cycles of activation for BRD4^FL^ −/+ JQ1. (H) PCC quantification and difference in PCC (deltaPCC) of overlaid images of 33 cells in subsequent activation cycles. Negative controls are PCCs between images of different cells. ***p = 0.0015 by Mann–Whitney exact, two-tailed *t* test. (I) Short-term (2 min) 200 nM JQ1 treatment disperses BRD4 condensates (JQ1), yet they form again quickly after washing out JQ1 (Recovery). Segmented masks of identified BRD4 puncta within nuclear outline, overlay of JQ1 and recovery timepoints (green) with pretreatment condensate positions (magenta). (J) Quantification of condensate dissolution during JQ1 treatment (shaded area) and recovery after washout. Error bars represent SD of three biological trials of 10 cells each. (K) Overlay of images before/during JQ1 treatment and before treatment/after JQ1 washout (left). PCC of 30 cells before/during JQ1, and before treatment/after JQ1 washout.

Because the chromatin-bound clusters of BRD4 exhibit some noncanonical condensation behaviors in phase diagrams, we sought to determine whether classical nucleation theory still applies to BRD4 condensate formation. If acetylated chromatin binding acts as a heterogeneous seed for BRD4 condensation, nucleation theory predicts that altered chromatin binding will manifest in changes to the following experimentally-accessible metrics: (1) the rate of condensate formation, (2) the delay time before visible condensates appear, and (3) the steady-state number density (number per unit area) of condensates (Figure 4, A and B). The third outcome arises because the growth of the first condensates to form suppresses the nucleation of additional condensates elsewhere (Hensley, Jacobs, and Rogers, 2022; Hensley *et al.*, 2023), and subsequent coalescence is extremely slow. Leveraging the high spatiotemporal control over condensate initiation enabled by the Corelet system, we measured these three parameters of BRD4^FL^ nucleation before and after addition of JQ1 (1 μm, 90 min). We find that disrupting acetylated chromatin binding leads to significantly lower nucleation rates, longer delay times, and ultimately a lower number density of condensates in JQ1-treated cells compared with the same cells before JQ1 treatment (Figure 4, C–E). These observations indicate that acetylated chromatin binding aids BRD4^FL^ condensate nucleation in this Corelet system assay.

To further test the concept that chromatin-binding BRD4 condensates are heterogeneously nucleated, we took advantage of the reversibility of the Corelet system to perform repeated activation-deactivation cycles of Corelet condensation and compared the nucleation positions of BRD4^∆N^ (Figure 4F) and BRD4^FL^ −/+ JQ1 (Figure 4G) condensates in subsequent cycles. Heterogeneous nucleation is a stochastic process biased toward forming condensates at certain seed locations, and thus should promote condensate formation preferentially at a set of nuclear locations over subsequent cycles, which would result in relatively high Pearson correlation coefficient (PCC) of overlaid images from multiple cycles, though inherent stochasticity will prevent the expected PCC from reaching 1. Conversely, homogeneous nucleation results in randomly located condensates that should exhibit lower PCC across cycles, representing randomly localized nucleation. As expected, we find low correlation between subsequent condensate positions of chromatin noninteracting BRD4^∆N^ (Figure 4F). Moreover, upon measuring PCC of BRD4^FL^ condensate nucleation sites in the same cell before and after JQ1 treatment, we find that condensates with acetylated chromatin-binding intact (−JQ1) exhibit higher correlation between nucleation positions in subsequent activation cycles than those without acetylated chromatin binding (+JQ1) (Figure 4, G and H). Interestingly, even the chromatin noninteracting +JQ1 case exhibits a slightly higher PCC than a negative control (measured by correlation with another cell), perhaps indicating anticorrelation with chromatin density as has been previously reported (Shin *et al.*, 2018; Lee *et al.*, 2023) but this effect is much smaller than the difference between −/+JQ1 experiments. These data suggest that acetylated chromatin-binding sites can act as nucleators of BRD4^FL^ condensation in living cells.

To ensure that these nucleation behaviors reflect acetylated chromatin binding and are not an artifact of Core particle binding, we investigated whether BRD4^FL^-miRFP condensates without the Corelet system form again at their initial positions after dispersal through JQ1 treatment (Figure 4I). We observed the nuclear position of BRD4^FL^-miRFP condensates before JQ1 treatment (pretreat), added JQ1 for 2 min, then washed out the JQ1 by refreshing the media twice in quick succession, and observed the dynamics and positioning of condensate reformation (Figure 4, I–K). Strikingly, condensates nucleate after JQ1 washout within seconds (Figure 4J) and have a strong correlation with their previous locations (Figure 4K), indicating that acetylated chromatin binding can heterogeneously seed BRD4 condensate nucleation without additional synthetic oligomerization through the Corelet system. Together, these results strongly suggest BRD4^FL^ condensates have preferential nucleation sites that are likely acetylated chromatin regions.

### Coarse-grained simulations characterize BRD4 chromatin-seeded nucleation behavior

**FIGURE 5:**
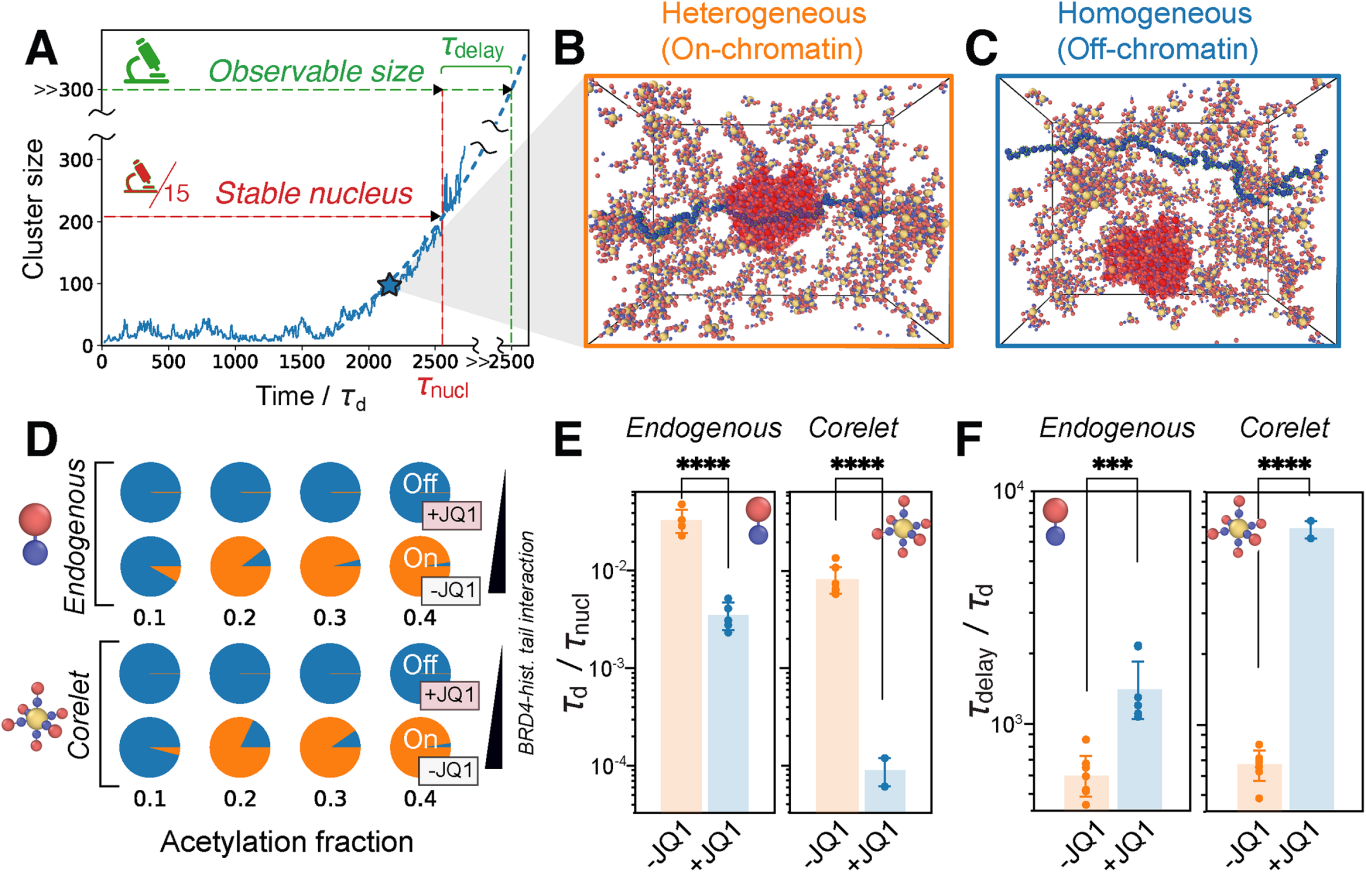
Simulations of BRD4^FL^ Corelet condensation quantify enhancement of chromatin-seeded nucleation. (A) Schematic of a typical nucleation event. *τ*_nucl_ is the time required for cluster size to reach the threshold to form a stable nucleus (red line). *τ*_delay_ is the time required for the nucleated cluster to grow via diffusion-limited growth to reach a size that is observable under the microscope (green line), which is estimated to be 15 times larger than the stable nucleus size in our simulations. (B) Example of a heterogeneous (on-chromatin) nucleation event, in which the largest cluster (highlighted in red) interacts with the chromatin polymer. (C) Example of a homogeneous (off-chromatin) nucleation event. (D) Probability of on-chromatin (orange) or off-chromatin (blue) nucleation events for endogenous (top) and Corelet (bottom) simulations at two BRD4-histone tail interaction strengths (representing +JQ1 and −JQ1), and four acetylation fractions (0.1, 0.2, 0.3, 0.4). (E) Quantification of the nucleation rate in endogenous and Corelet simulations with (−JQ1) and without (+JQ1) strong BRD4-histone tail interactions. (F) Quantification of the delay time before observable condensates are formed in endogenous and Corelet simulation systems with (−JQ1) and without (+JQ1) strong BRD4-histone tail interactions. (E, F) Seven independent simulations for each measurement are run at an acetylation fraction of 0.4 for both +JQ1 and −JQ1. In the +JQ1 Corelet simulations, only two out of seven simulations achieved nucleation. Error bars represent the SE. ***p ≤ 0.001, ****p ≤ 0.0001 by Student’s *t* test.

Utilizing the coarse-grained simulation system introduced in Figure 3, we can explore the molecular scale parameters that underlie the experimentally observed change in BRD4 nucleation behavior with and without acetylated chromatin binding. A typical nucleation event in a constant-pressure simulation is characterized by following the time-dependent evolution of the largest cluster of condensing species (Figure 5A). Simulation time is measured with respect to the time required for a Corelet to diffuse its own diameter, *τ*_d_. Initially, small clusters form and dissolve, until a cluster larger than the critical nucleus emerges stochastically and grows to form a stable condensate, *τ*_nucl_. This cluster will then continue to grow until it is large enough to be visualized in experiments after a time *τ*_delay_.

We expected that BRD4 condensate nucleation should depend on (1) supersaturation, (2) acetylated chromatin-binding capability, and (3) the fraction of chromatin tails that are in an interaction-competent state. To this end, simulations were performed with a variable fraction of chromatin tails in the interaction-competent state, and with the condensing species chosen to be either the BRD4^FL^ Corelet (valence = 6) or the BRD4^FL^ endogenous monomer, at a series of supersaturation degrees, *S*. This BRD4^FL^ Corelet valence of 6 is above the critical valence required for off-chromatin nucleation, such that both heterogeneous nucleation (on-chromatin, Figure 5B) and homogeneous nucleation (off-chromatin, Figure 5C) are possible, depending on the simulation conditions.

In simulations performed with exceedingly weak attractive forces between the interaction-competent tails and BRD4, paralleling JQ1-treated cells, off-chromatin nucleation occurs in 100% of simulations regardless of the fraction of interaction-competent tails (Figure 5D, low chromatin interaction). However, if the simulations are performed with strong interactions between BRD4 and histone tails, paralleling control (−JQ1) conditions, chromatin-seeded nucleation occurs with higher probability at higher acetylation levels (Figure 5D, high chromatin interaction strength). Remarkably, with acetylated chromatin interaction, the transition from off- to on-chromatin nucleation is a sharp function of the fraction of acetylated histone tails and occurs between 10 and 20% of tails acetylated (Figure 5D). This implies that, on average, only one acetylated histone tail per nucleosome is necessary to shift to a majority of condensates nucleating on-chromatin in both the Corelet and endogenous BRD4 models.

We expected from classical nucleation theory that the ratio of off- to on-chromatin nucleation would also depend on the degree of supersaturation *S*, with increased *S* leading to more off-chromatin nucleation. To confirm this expectation, we repeated our simulations of the endogenous BRD4 and Corelet systems at 3 degrees of supersaturation (Supplemental Figure S3A). As predicted, higher supersaturation leads to more off-chromatin nucleation, and this effect is stronger with the Corelet system, due to the extra oligomerization from the Core platform.

To compare our simulations with the experimentally accessible metrics of nucleation in Figure 4, we estimated the nucleation rate, 1/⟨*τ*_nucl_⟩, and average delay time, ⟨*τ*_delay_⟩, for both models under highly acetylated conditions with either weak BRD4–chromatin interactions (+JQ1) or strong BRD4–chromatin interactions (−JQ1) (Figure 5, E and F). We find that, similar to experiments, the delay time is increased and the nucleation rate is decreased without chromatin binding for both Corelets and endogenous BRD4 model systems. Supersaturation also affects *τ*_nucl_ and *τ*_delay_, with higher supersaturation leading to lower nucleation rates and longer delay times (Supplemental Figure S3, B and C). Together, these simulations state that acetylated chromatin is a potent nucleator of BRD4 condensation, that the rate of nucleation depends on the fraction of acetylated tails, and that the influence of chromatin interactions on the nucleation dynamics are highly similar for endogenous BRD4 condensation and multivalent Corelet-driven BRD4 condensation.

### Chromatin acetylation controls localized BRD4 condensation

Given that both our coarse-grained simulations and experiments suggest that acetylated regions can act as preferential nucleation sites, altering the amount of acetylated chromatin should affect the nucleation behavior of BRD4 condensates. In particular, our simulations predict that reducing acetylation levels below one acetyl per nucleosome should increase the probability of nucleating off-chromatin via the slower homogeneous pathway (Figure 5D). To manipulate the acetylation in living cells, we treated cells with two drug treatment paradigms: 1 μM A485 for 24 h, which inhibits p300 histone acetyltransferase and decreases the H3K27 acetylation level to half that of Wild-type levels, or 100 nM trichostatin A (TSA) for 24 h, which inhibits histone deacetylases thereby increasing H3K27 acetylation level by 3.2-fold (quantified by immunofluorescence in Supplemental Figure S4, A and B). While BRD4’s bromodomains are capable of binding a variety of acetylated marks including H3K9/27Ac and H4K5/8/12/16Ac, its binding to H3K27Ac (Zeng and Zhou, 2002) is well established so we use this as a marker for overall acetylation in this section (Jiang *et al.*, 2020).

**FIGURE 6:**
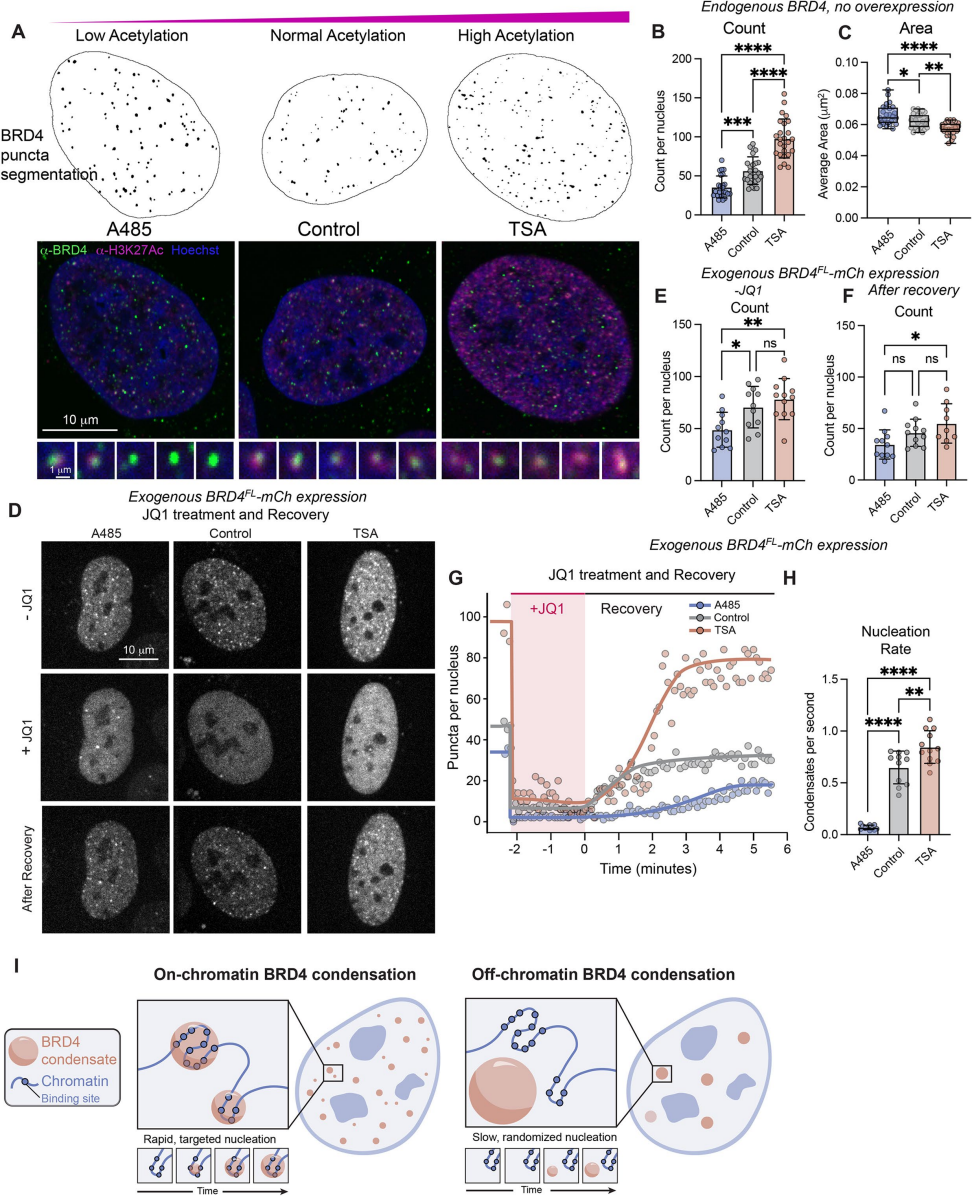
Epigenetic acetylation level influences nucleation behaviors in endogenous and exogenous BRD4 systems. (A) Representative images and analysis examples of endogenous BRD4 puncta by immunofluorescence in cells treated with control media (Control), histone acetyltransferase inhibitor (A485), or histone deacetylase inhibitor (TSA). (B, C) Quantification of endogenous BRD4 condensate count per nucleus (B) and condensate average area (C) measured by immunofluorescence in drug-treated cells. *n* = 25 cells each, error is SD. ***p = 0.002, ****p = 0.0001 by one-way ANOVA. **D.** Representative images of BRD4^FL^-mCh puncta in living cells treated with control media, A485, or TSA at three timepoints: before addition of JQ1 (−JQ1), during JQ1 incubation (+JQ1) and after JQ1 washout and recovery. (E–F) Quantification of the number of condensates per nucleus in drug-treated cells before addition of JQ1 (E) and after washout and recovery (F). Expression range as defined in Supplemental Figure S4, D and E. *n* = 11, 11, 12 cells, error is SD, *p = 0.031, **p = 0.008 by one-way ANOVA. (G) Timecourse graph of the number of BRD4^FL^-mCh puncta per nucleus during JQ1 treatment (shaded area) and nucleation after washout. (H) Nucleation rate of BRD4^FL^-mCh puncta measured as slope after JQ1 washout. *N* = 11, 11, 12 for A485, control, TSA respectively. **p = 0.006, ****p = 0.001. (I) Model figure summarizing findings about on- and off-chromatin BRD4 nucleation.

Immunofluorescence of endogenous BRD4 at high resolution shows nuclear BRD4 colocalizing with regions of H3K27Ac (Figure 6A, control). Upon A485 treatment, the BRD4 puncta appear larger and less colocalized with H3K27Ac, potentially representing an increase in off-chromatin nucleation. Increased amounts of H3K27Ac are clearly visible in TSA-treated cells and exhibit high colocalization with BRD4. The number of puncta per nucleus in this endogenous case represents the number density after nucleation. Quantification shows that the BRD4 puncta in A485-treated cells with lowered acetylation are less abundant (Figure 6B) and larger (Figure 6C) than control cells, while the BRD4 puncta in TSA-treated cells that have increased acetylation are more abundant and smaller than control, even though the endogenous expression level of BRD4 remains unchanged across these conditions (Supplemental Figure S4C). This observation is consistent with a shift in the ratio of on-chromatin and off-chromatin nucleation toward off-chromatin in the lowered acetylation case, and toward on-chromatin in the increased acetylation case.

To directly measure the nucleation rate of BRD4^FL^-mCh condensates in TSA- and A485-treated cells without extra oligomerization from Corelets, we utilized application and washout of JQ1 (Figure 6, D–H, similar to Figure 4J). In agreement with the altered number of endogenous BRD4 puncta, we observe a significantly decreased number of BRD4^FL^-mCh condensates in live A485-treated cells with lowered acetylation, and an increased number of puncta in TSA-treated cells with higher acetylation, though this does not reach significance (Figure 6, D and E). Addition of 1 μM JQ1 disrupts chromatin binding and results in a rapid decrease in the number of condensates in all cells (Figure 6, D and G). After 2 min of JQ1 treatment, the media was washed twice in quick succession to remove the JQ1 and the rate of BRD4^FL^-mCh condensate nucleation and final number density of condensates postrecovery were recorded (Figure 6, F and G). Fitting the linear portion of the slope reveals that A485-treated cells with lower acetylation nucleated BRD4 condensates significantly slower than control, and TSA-treated cells with increased acetylation nucleated significantly faster (Figure 6H), consistent with the predictions made by our simulations. Notably, the number density difference between A485 and TSA-treated conditions in cells exogenously expressing BRD4^FL^ is less significant than the endogenous number density difference. This likely arises due to overexpression increasing the supersaturation and thereby making the system inherently less dependent on chromatin as a nucleation site.

Collectively, these results demonstrate how substrate binding through epigenetic marks can promote heterogeneously seeded condensate formation, underscoring the importance of epigenetic control over chromatin-bound condensate targeting through modulation of epigenetically modified chromatin valence. Altogether, we demonstrate that epigenetic modification of chromatin allows for rapid condensation of transcriptional activators like BRD4 at specific chromatin sites.

## DISCUSSION

Here we described the biophysical impact of chromatin binding on condensate formation by BET family protein BRD4, which is biologically implicated in potent transcriptional activation, and is commonly misregulated in cancer (Donati *et al*., 2018; Mehta and Zhang, 2022; Tang *et al.*, 2022). We set out to investigate targeting of condensation using BRD4 as an example of a condensation-prone protein that also binds to acetylated chromatin. We showed that BRD4’s binding to the acetylated chromatin substrate enhances both kinetic and thermodynamic properties of BRD4 condensation in three experimental systems: endogenous BRD4 distribution, exogenous BRD4-mCherry expression, and synthetically oligomerized BRD4-mCherry-sspB in the Corelet system. We also developed coarse-grained simulations that represent condensation in both the Corelet and endogenous systems, which allowed quantitative characterization of varying valence, supersaturation, and acetylation fraction on condensation. With this coarse-grained model, we were able to create phase diagrams that matched the valence shift observed upon loss of acetylated chromatin binding in Corelet experiments. This also revealed that the presence of these chromatin-interacting clusters does not delineate a unique binodal boundary. Additionally, in this low-valence regime, the size of the cluster depends on the length and extent of the acetylated region, since chromatin is a necessary, often majority, component of the cluster under these conditions. This represents a potential mechanism of controlling the size of BRD4 clusters at low expression levels, that is in agreement with experimentally observed BRD4 condensate size distribution (Supplemental Figures S2 and S4).

Even though chromatin-bound BRD4 creates noncanonical condensates with chromatin as a major component at low valence, we found that nucleation of these condensates was still consistent with the principles of classical nucleation theory through heterogeneous nucleation. Indeed, acetylated chromatin regions can act as seeds for heterogeneous nucleation of BRD4 condensates, providing rapid and targeted condensate formation at sites of epigenetic acetylation. With an overall acetylation fraction of 20%, simulated BRD4 condensates nucleate almost exclusively on-chromatin, meaning that acetylated chromatin is a potent nucleation substrate and that we expect almost all BRD4 condensates in living cells to be associated with an acetylated chromatin region. In agreement with this, repeated activation-deactivation cycles of Corelet condensation demonstrated that BRD4 condensates repeatedly form at preferred sites on chromatin, and non-Corelet BRD4-mCherry condensates nucleate again at specific locations after disruption of chromatin attachment through JQ1 treatment. Relatedly, we find that manipulation of acetylation level through epigenetic inhibitors affects the average nucleation rate of BRD4-mCherry exogenous expression condensates, as well as the number density of endogenous BRD4 droplets. These data provide a mechanism for selective formation of BRD4 condensates at acetylated regions and, excitingly, are consistent with changes to endogenous BRD4 distribution through altered nucleation pathways.

Historically, models of transcription factor targeting have been put forth that suggest mechanisms by which chromatin-binding proteins find their cognate binding sites in the large volume and complex environment of the eukaryotic nucleus (Lu and Lionnet, 2021). These models commonly focus on describing diffusion pathways for individual molecules that reduce the dimensionality of the system to enable a more efficient single-molecule search for a rare binding site (Jana, Brodsky, and Barkai, 2021). Here, we propose a complementary model of heterogeneously seeded transcriptional coactivator condensate nucleation (Figure 6I), in which BRD4 condensates are rapidly heterogeneously seeded at chromatin regions enriched with acetylated binding sites, resulting in many targeted but size-limited condensates per nucleus. We emphasize that when acetylation levels are heterogeneous, as they are in the nucleus, the minimal conditions for the formation of finite-size condensates are predicted to correspond to the most highly acetylated regions of chromatin. Disrupting chromatin binding through JQ1 treatment or overexpression of BRD4 can lead to off-chromatin nucleation, which occurs more slowly at random nuclear locations, and results in fewer, larger, and randomly localized condensates. This model explains both targeted binding of transcriptional activators to chromatin-localized binding sites, and depleted nucleation at off-target sites after on-target sites have nucleated. It is also consistent with published data on BRD4 targeting being dependent on its expression level (Maruyama *et al.*, 2002; Liang *et al*, 2021; Wang *et al.*, 2021; Drumond-Bock *et al.*, 2023) and chromatin acetylation patterns (Alekseyenko *et al.*, 2017; Donati *et al.*, 2018; Sudarshan *et al.*, 2022).

Our condensation model of BRD4 chromatin targeting provides context for altered BRD4 chromatin localization patterns observed in disease states due to aberrant acetylation patterns (French *et al.*, 2003; Lovén *et al.,* 2013). Interestingly, the BETi JQ1 was originally developed as an anticancer therapy for tumors of various origins to prevent transcriptional expression of oncogenes that become hyperacetylated (Filippakopoulos *et al.*, 2010). We found that JQ1 treatment disrupts chromatin binding, as expected, but does not completely abrogate BRD4 condensation ability—instead, JQ1 treatment leads to randomized localization of BRD4 nucleation. Similarly, other small-molecule drugs that target chromatin-interaction motifs may modulate but not abrogate condensation of transcription factors, and therefore lead to variable biological effects.

The modulation of condensation behaviors through chromatin binding that we observe with BRD4 may represent a general phenomenon that applies to other BET family proteins or other transcription factor families. We expect that transcription factors with dual chromatin binding and condensation capabilities, similar to the dual structure of BRD4, will follow the kinetic and thermodynamic paradigms described here, including chromatin-bound clustering at low valence and concentration, as well as heterogeneously seeded nucleation at multivalent chromatin binding regions. Additionally, because many transcription factors and coactivators interact with one another, it will be interesting in future studies to investigate how condensation and chromatin binding of binding partners influence one another, for example BRD4 and MED1 (Sabari *et al.*, 2018). Overall, our results put forth a model in which the cell utilizes epigenetic modifications to regulate condensation properties of transcriptional activators in order to target their nucleation and enact a specific gene expression profile.

## MATERIALS AND METHODS

Request a protocol through *Bio-protocol*.

### Cell culture

U2OS cells, obtained from the ATCC and authenticated using ATCC’s short tandem repeat profiling, were cultured in DMEM (GIBCO, 11995065) supplemented with 10% FBS (Atlanta Biological, S11150H) and 1% streptomycin and penicillin (GIBCO, 15140122). The cells were grown under conditions of 37°C with 5% CO_2_ and tested for mycoplasma regularly.

### Immunofluorescence

Cells plated in 96-well plates were fixed by adding 4% paraformaldehyde for 10 min. Following fixation, cells were subjected to a 5-minute wash with buffer (0.35% Triton-X, Thermo Fisher Scientific PRH5142, in PBS) and permeabilized with 0.5% Triton-X in PBS for 1 h. Subsequently, cells were blocked for 1 h using blocking buffer (0.25% Triton-X, 5% FBS, in PBS). Primary antibodies (anti-BRD4, Cell Signaling Technology mouse mAb #63759 at 1:1000 dilution, anti-H3K27ac, Active Motif #39133, 1:1000 dilution) were prepared in blocking buffer and incubated on the sample overnight at 4°C. The following day, cells were washed three times for 5 min each with washing buffer. The secondary antibodies (AlexaFluor 647 goat-anti-rabbit Thermo Fisher Scientific A-21245, 1:1000 dilution) were prepared in blocking buffer and incubated for 2 h at 4°C. Cells were then washed three times for 5 min each with wash buffer, followed by a 20-minute incubation with Hoechst (1:2000 dilution). Finally, cells were washed with PBS.

### Construct design and cloning

The full-length BRD4 DNA fragment was amplified by PCR from pcDNA4-TO-HA-BRD4FL (Addgene plasmid #31351) (Rahman *et al.*, 2011) incorporated into linearized FM5 lentiviral vectors containing standardized linkers (generously provided by David Sanders) using the In-Fusion HD cloning kit (Takara Bio, 638910). BRD4^dN^-mCh-sspB (Addgene plasmid #121968) (Shin *et al.*, 2018) and NLS-iLID-Ferritin (Addgene plasmid #122147) (Bracha *et al.*, 2019) were originally developed and characterized in previous Brangwynne lab studies. The validity of all constructs was verified through GENEWIZ (Azenta Life Sciences) plasmid Sanger sequencing.

### Lentivirus production and lentiviral transduction

For all live-cell experiments, cells were stably transduced with lentivirus. Lentiviruses were generated by seeding Lenti-X 293T cells (Takara Bio, catalogue no. 632180) in 6-well plates, at ∼70% confluence at the time of transfection. After 24–48 h, the transfer plasmid and helper plasmids VSV-G and PSP (generously provided by David Sanders) were transfected into the Lenti-X cells using Transit293 transfection reagent (Mirus, catalogue no. MIR 2700) and incubated in OptiMEM (Thermo Fisher Scientific, catalogue no. 31985062). Approximately 48 h after transfection, the viruses were harvested and filtered using a 0.45-μm filter (VWR, catalogue no 28144-007). The resulting viruses were either used immediately or stored at −80°C. U2OS cells were plated in 96-well glass-bottom plates (Thermo Fisher Scientific, catalogue no NC0536760) at 30–50% confluency and transduced with lentivirus for 2–3 d prior to live-cell imaging experiments.

### Drug treatments

JQ1 (MedChemExpress, catalogue no. HY-13030) diluted in DMSO was added to the cells to a final concentration of 1 μM, and incubated for 90 min prior to imaging. The +JQ1 cells were imaged in media containing JQ1. TSA (Sigma, T8552-1MG) was resuspended in DMSO and added to the cells to a final concentration of 100 nM, and incubated for 24 h prior to imaging. A485 (MedChemExpress, catalogue no. HY-107455) was resuspended in DMSO and added to the cells to a final concentration of 200 nM, and incubated for 24 h prior to imaging.

### Corelet system optogenetic activation

Cells expressing Corelet system constructs (NLS-iLID-GFP-Ferritin and either BRD4^dN^-mCh-sspB or BRD4^FL^-mCh-sspB) via lentiviral transduction were seeded on 96-well glass bottom plates (Thermo Fisher Scientific, catalogue no NC0536760), then loaded onto a Nikon A1 laser scanning confocal microscope with Nikon Eclipse Ti2 body. A 100X oil immersion Apo TIRF objective (NA 1.49) was used to visualize these samples. Obtaining images with the 488 nm laser at 0.1% laser power and above is sufficient to trigger iLID–sspB interaction and Corelet system optogenetic activation. Activation movies were obtained with 488 nm and 512 nm lasers every 5 s for 3 min.

### Image analysis for count, size, total area, and volume of puncta per nucleus

Image analysis was performed in Fiji (Schindelin *et al.*, 2012), an open-source image analysis platform. Nuclei were initially segmented from single z-plane images of BRD4^FL^-mCh alone (Figure 1) or from the final frame of Corelet activation movies (Figure 2) using the mCherry (561 nm laser) channel and an Otsu thresholding method, with smoothing if necessary to obtain a nuclear mask. Puncta were segmented within each nucleus using the IsoData method in Fiji after smoothing and rolling ball background subtraction with a radius of 4 pixels. The Analyze Particles feature was used to measure the area, count, and intensity of pixels within puncta for each nucleus. Graphs of puncta Count were created by averaging the number of puncta per nucleus across 25 nuclei within a well (biological replicate), then across four biological replicates. From the same data, puncta per nucleus were also plotted on a cell-by-cell basis on a log-log plot before and after addition of JQ1 in the same nucleus (Supplemental Figure S2D). Graphs of puncta average size were created by first averaging the average area of puncta within a nucleus, then across 25 nuclei within a well (cells within one well consisting of a biological replicate), then across four biological replicates. Condensate volume was estimated by raising the average area of condensate to the power of 3/2, then multiplying this by the number of condensates per nucleus.

### Phase diagram data acquisition

The Nikon A1 laser scanning confocal microscope is equipped with PicoQuant software and was calibrated by FCS at a range of 488 nm and 561 nm laser settings from 0.1 to 5% power at 512 × 512 or 1024 × 1024 pixels per image to create a quantitative conversion from fluorescence arbitrary units (AU) of GFP and mCherry fluorophores to micromolar concentrations of these proteins. U2OS cells expressing Corelet system components (NLS-GFP-iLID-Ferritin and BRD4^FL^-mCh-sspB) were loaded onto the Nikon A1 laser scanning microscope. To ensure consistency, cells were allowed to equilibrate to stage temperature for 30 min before imaging, and a standardized imaging protocol was applied to all cells used in the phase diagram. Activation movies were obtained every 2 s for 3 min with 488 nm and 512 nm lasers. Positions of acquired cells were marked, then media containing JQ1 to a final concentration of 1 µM was added to the imaged well and incubated for 90 min on the stage top. A second round of activation series of images every 2 s for 3 min were taken of all cells, used for +JQ1 data.

### Corelet system image analysis and phase diagram construction

Only nuclei fully within the field of view were included in the analysis. Nuclei were segmented using ImageJ Otsu method, and the average fluorescence intensity of GFP and mCherry was measured based on the first frame of the movie, before activation. The intensity measurement in AU was converted to micromolar concentration via the calibrated imaging settings. Cells were marked as “Yes PS” or “No PS” through qualitative assessment of the first and last frames of the 3-minute activation series, determined by whether new condensates were formed during the activation period. Qualitative assessments were conducted by two independent observers, with one observer blinded to the experimental conditions. The evaluations of the two observers showed high consistency in almost all cases. Instances where the observers disagreed on cell classification were excluded from the results. Core concentration for each nucleus was calculated as the GFP concentration divided by 24 (as there are 24 GFP monomers for each assembled Ferritin Core). Valence of each nucleus was calculated as the ratio of sspB-fused protein (mCherry concentration) to Core (GFP concentration of 24 mers). Data were plotted in GraphPad Prism 10, and shaded area was added manually in Adobe Illustrator to guide the eye.

### Nucleation measurements

U2OS cells expressing Corelet system components (NLS-GFP-iLID-Ferritin and BRD4^FL^-mCh-sspB) were imaged on a Nikon X1 spinning disk confocal (described above) in 488 and 561 channels every 2 s for 5 min. The number of condensates per nucleus were counted in each frame through image analysis in Fiji, with smoothing and rolling ball background subtraction radius 4, then thresholded with IsoData algorithm and counted per nucleus using the Analyze Particles feature.

#### Slope, delay time, and number density.

Nucleation slope was determined by fitting the rate of puncta per nucleus formed over the linear portion of the curve. For −JQ1 samples, the linear portion of the curve usually consisted of the first three to four frames and for +JQ1 samples, the linear portion was generally shifted later. Delay time was determined as the time in seconds before the number of puncta increased consistently for two frames. Number density was determined as the count of puncta per nucleus at the last frame of the activation series (5 min of activation).

### Repeated cycles of Corelet activation and deactivation

U2OS cells expressing Corelet system components (NLS-GFP-iLID-Ferritin and BRD4^FL^-mCh-sspB) were imaged on a Nikon X1 spinning disk confocal (described above) for five segments: Activation-Deactivation-Activation-Deactivation-Activation. “Activation” with 488 and 561 channels imaged every 2 s for 3 min, “Deactivation” with only 561 channel imaged every 2 s for 5 min (absence of 488 nm laser allows deactivation of iLID–sspB interaction).

#### PCC.

Activation-deactivation cycling series were registered in Fiji using HyperStackReg, Rigid Body, to account for cell movement. The last frame of each of three activation cycles of the registered series was extracted, and PCC was run between 4 × 4 micron square areas of the same cell (experiment) and different cells (negative control) using the JaCoP plugin in Fiji. Correlation between cycles 1 and 2 and cycles 2 and 3 are both reported.

### JQ1 washout experiments

U2OS cells were lentivirally transduced with BRD4^FL^-mCh-sspB, plated on 96-well glass-bottom plates and imaged 48–72 h after transduction. Plates were loaded onto a Nikon spinning disk microscope (Yokogawa CSU-X1) on a Nikon Eclipse Ti body and DU-897 EMCCD camera and LU-NV laser launch, with stage top incubation of 37°C and 5% CO_2_ conditions maintained by Okolab microscope stage incubator with 96-well insert. A 100X oil immersion Apo TIRF objective (NA 1.49 MRD01991) was used to obtain images with 488 nm and 561 nm lasers to visualize GFP and mCherry constructs, respectively. Movies were obtained once in each well at a frame rate of one image every 5 s for 10 min, with the first three frames (15 s) as “pretreatment” to establish the initial number of condensates per nucleus. After the first three frames, media containing JQ1 was added to the open top of the well to a final concentration of 100 nM JQ1 and cells imaged for 24 frames (2 min), called “JQ1 treatment.” After 2 min of JQ1 treatment, the media was removed and replaced with fresh media (no drug) twice in quick succession, then imaged until a total of 10 min, during which time the BRD4 condensates nucleated again, called “recovery.” This process was repeated in each well, with cells in the same frame considered technical replicates and cells in different wells considered biological replicates.

#### Washout PCC.

JQ1 washout series images were registered to correct for cell movement using HyperStackReg in Fiji. Frames from the end of JQ1 treatment and end of recovery were compared in the JaCoP plugin for “during JQ1” PCC, and frames from the pretreatment and end of recovery period were compared for “Recovery” PCC.

### Simulations

#### Coarse-grained model.

The model for a BRD4 protein consists of two blobs representing the globular domain-containing (N-terminal) and disordered (C-terminal) portions of the protein. Corelets are constructed by attaching BRD4 molecules to a particle representing the ferritin core. Differential valence is represented by modifying the number of BRD4 molecules attached to the core particle in each simulation. Chromatin is represented by a chain of nucleosome particles, each of which is decorated with eight small blobs representing the histone tails. Histone tails were each assigned one of two states: a non-acetylated state, in which they do not interact with BRD4, or an acetylated state, in which they are highly attractive to the N-terminal blob of a BRD4 molecule.

There are three main types of interactions in the system:

1. Steric repulsion is represented by a WCA repulsive pair potential (Weeks, Chandler, and Andersen, 1971). For the BRD4 N-terminal, C-terminal, and histone tail blobs, the WCA diameters are chosen to enforce a maximum packing fraction consistent with the total volume occupied by the amino acids comprising each of the blobs. For the ferritin core and histone particles, the WCA diameters are chosen based on structural data.
2. Bonds between nucleosomes, between the N- and C-terminal polymer blobs of BRD4, and between BRD4 molecules and the oligomerization platform cores are represented using FENE potentials (Kremer and Grest, 1990). Spring constants and equilibrium bond lengths for bonds between polymer blobs are chosen based on an ideal polymer model.
3. Interactions between polymer blobs are modeled using the Flory-Krigbaum potential (Flory and Krigbaum, 1950; Lenart, Jusufi, and Panagiotopoulos, 2007; Jusufi, Hynninen, and Panagiotopoulos, 2008), which describes polymers in dilute solution in terms of their radii of gyration and a variable strength of the interaction. Radii of gyration are estimated based on an ideal polymer model, and interaction strengths are chosen to reproduce the experimental phase diagram.

All molecular dynamics (MD) simulations were run using the LAMMPS package (Plimpton, Thompson, and Wood, 2021).

#### Phase diagram calculations.

The measurement of coexistence densities and the determination of phase separation in the presence or absence of chromatin was accomplished through direct coexistence simulations. All simulations were performed in the NVT ensemble with the Nosé–Hoover thermostat and used a 120 × 120 × 561 nm^3^ orthogonal box with periodic boundary conditions in all three directions. For simulations without chromatin, 343 BRD4^FL^ Corelets were set up to be in slab geometry, which is a liquid (dense) phase slab in the x-y plane, so as to create two flat interfaces. For simulations with chromatin present, slab systems with 343, 86, and 43 Corelets were created to test multiple Corelet densities. Chromatin was constructed as a 400-nucleosome chain along the *z*-axis, passing through the liquid (dense) phase slab of Corelets. The opposite ends of the chromatin were bonded to each other through the *z*-axis periodic boundary. Measurements of coexistence densities in the absence of chromatin were obtained by analyzing the distribution of Corelets across the *z*-axis, from which the densities in the dilute and dense regions could be determined. To identify phase separation in the presence of chromatin, we devised a quantitative definition for detecting finite-size condensates based on the distribution of Corelets and chromatin along the *z*-axis. Specifically, finite-size condensates were detected whenever the maximum and minimum densities along the *z*-axis of either the Corelets or the nucleosomes were found to differ by at least 3-fold. This definition accounts for the enrichment of Corelets and the compaction of chromatin in finite-size condensates across the full range of Corelet concentrations probed in Figure 3C.

#### Nucleation simulations.

Nucleation simulations were conducted with periodic boundary conditions, where the z-direction is percolated by periodic chromatin. The x and y-directions were held at constant pressure to enforce approximately constant supersaturation in the process of condensation. The simulation box dimension in the z-direction was kept fixed to avoid unphysical coupling of the chromatin tension to fluctuations in the other box dimensions. Langevin dynamics were then used to account for an implicit solvent. Nucleation simulations were conducted using either 512 Corelets or 5832 endogenous BRD4 molecules. The number of simulated nucleosomes varies in the range ∼330–360 depending on the supersaturation. Nucleation was monitored by clustering all molecules based on a cut-off distance, *R*_clust_, and tracking the number of molecules in the biggest cluster, *N*_CS_. The cut-off distances of *R*_clust_ = 25 nm for Corelets and 12.5 nm for endogenous BRD4 were chosen based on the distance at which the nonbonded interactions between pairs of molecules are negligible for any molecular orientation.

Nucleation transition paths are defined as the first-passage portion of a trajectory in which the largest cluster in the system grows to become a stable droplet. Specifically, a transition path begins when *N*_CS_ increases beyond fluctuations around the initial metastable state and passes directly to a stable cluster size of ∼200 Corelets or ∼600 BRD4 molecules. These stable cluster sizes correspond to ∼1/15 and 1/75 of the smallest registerable droplet sizes in the experiments, as determined from the experimental pixel dimensions and the condensed-phase densities obtained from equilibrium simulations. The first passage time, *τ*_nucl_, is recorded as the time required to reach the stable droplet size, and the nucleation rate is defined as *J* = 1/⟨*τ*_nucl_⟩. The delay time *τ*_delay_ is obtained from the intersection of the extrapolated transition path, assuming a diffusion-limited growth law 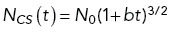 (Ham, 1958), and the smallest registerable droplet size in the experiments. On-chromatin and off-chromatin nucleation and growth regimes were distinguished based on the average chromatin coverage by the largest Corelet or BRD4 cluster in the system, which is computed based on a cut-off distance of *R*_cut_ = *R*_clust_ = 25 nm between nucleosomes and Corelet clusters or 12.5 nm between nucleosomes and BRD4 clusters, respectively.

### Code availability

All simulations were performed using the LAMMPS molecular dynamics package, which is publicly accessible.

## Supplementary Material



## Abbreviations used:

BD: bromodomain
BET: bromodomain and extra-terminal
BETi: BET protein inhibitor
CTM: C-terminal motif
ET: extra-terminal
GFP: green fluorescent protein
H3K27Ac: Acetylation on histone H3 lysine 27
IDR: intrinsically disordered region
MD: molecular dynamics
miRFP: monomeric infrared fluorescent protein
PCC: Pearson Correlation Coefficient
PS: phase separation
TSA: trichostatin A
WT: Wild-type
