# Supplementary material for: Interplay of condensation and chromatin binding underlies BRD4 targeting
Source: Mol Biol Cell. 2024 May 21;35(6):ar88. doi: 10.1091/mbc.E24-01-0046 (PMC11238092; doi:10.1091/mbc.E24-01-0046)
Supplement: Supplementary file 2 [file mbc-35-ar88-s001.pdf]

Supplemental Materials

Molecular Biology of the Cell

Strom *et al*.

Figure S1. JQ1 does not affect BRD4$^{\Delta N}$ puncta, related to Figure 2

A. Schematic diagram of the Corelet system, light-induced oligomerization platform that triggers droplet formation of phase-separation-prone protein regions in living cell nuclei. **B.** Representative images of BRD4$^{\Delta N}$ light-induced Corelet-condensates +/- JQ1 treatment. Quantification of count (**C**) and area (**D**) of BRD4$^{\Delta N}$ Corelet condensates +/- JQ1 in 3 trials of 25 cells each. Student's t-test. **E.** Single-cell BRD4^{FL} Corelet-condensate count per nucleus (pink) before JQ1 treatment (X-axis) and after JQ1 treatment (Y axis). Each point is one nucleus. Number of BRD4$^{\Delta N}$ Corelet condensates per nucleus (black) are unaffected by JQ1 and lie on the diagonal. Quantification of count (**F**) and area (**G**) of BRD4^{FL} condensates in cells expressing Corelet components but without light activation. Error bars represent SEM across four trials of 25 cells each, **** p = 0.0001 by Student's t-test.

Figure S2. Valence-dependence of BRD4 Corelet condensate volume, related to Figure 3

A-B. Experimental phase diagrams -/+ JQ1 recolored by average volume of condensed phase per nucleus. **C.** Phase diagram condensate volume data from -/+JQ1 cells plotted with volume as the independent variable. Note that clusters below valence^{-1} 1/5 are very small, consistent with simulation predictions. (-JQ1 n = 16, 37, 36, 41, 14; +JQ1 n = 16, 36, 37, 12, 0 cells in valence bins 1/32, 1/16, 1/8, 1/4, 1/2, respectively). Error bars SD. **D.** Simulation measurements of fraction of corelets in liquid (dense) phase, demonstrating that below the critical valence (dotted line), clusters can form but no stable dilute fraction is achieved. **E.**

Representative segmentations of nuclear outlines (blue) and puncta (black), with marked Yes PS (Y) / No PS (N) calls from a field of BRD4^{FL} Corelet cells in -JQ1 (top) and +JQ1 (bottom) conditions, before light activation (Pre-act, left), and during light activation (Act., right). Y* is a nucleus called as 'Yes PS' in -JQ1 that entered mitosis before the +JQ1 images were taken and so was excluded from the analysis. See also Movie S1. **F.** Simulation Corelet BRD4FL phase diagrams with chromatin at three acetylation levels; 20%, 30% and 40% of tails acetylated. The critical Corelet valence for the formation of finite-size chromatin-associated condensates depends on the fraction of acetylated histone tails in the coarse-grained simulations.

Figure S3. The ratio of homogeneous to heterogeneous nucleation is dependent on supersaturation, related to Figure 5

A. Pie charts representing the probability of on- (orange) and off-chromatin (blue) nucleation in Endogenous and Corelet simulation systems at three supersaturation levels (highest at top). Supersaturation, *S*, is defined as the ratio of the applied pressure to the equilibrium coexistence pressure. **B.** Nucleation rates and **C.** delay times as a function of the supersaturation. Error bars represent the standard error.

Figure S4. Epigenetic modifying drugs alter acetylation but not BRD4 expression level, related to Figure 6

A. Representative images of immunofluorescence of U2OS cells treated with DMSO (control), 1 uM A485, or 100 nM TSA for 24 hours, stained with antibodies that recognize BRD4 (green) and H3K27Ac (magenta). **B.** Quantification of H3K27 Acetylation intensity across 3 biological replicates of 25

cells each. Error is SD, ****p=0.0001 by one-way ANOVA. **C.** Quantification of BRD4 endogenous expression level across 3 biological replicates of 25 cells each. Error is SD. ns = not significant by one-way ANOVA. **D.** Count of condensates per nucleus as a function of BRD4^{FL}-mCh expression level in living cells. An expression level gate is used to bound the expression level of cells for calculating average count in Fig. 6E, the same as was used in Fig. 1. **E.** Nucleation rate as a function of expression level is shown, with the expression level gate shaded.

Supplementary Information: Coarse-grained simulation parameters

In this section, we describe the coarse-grained force field and the simulation environment. Parameter values are provided in the tables below.

Non-bonded interactions between particles/blobs of types 1 and 2 are modeled via a combination of a repulsive WCA potential[S1] and an attractive Gaussian potential. For the WCA interactions, the length scale is $\sigma_{12} = (\sigma_1 + \sigma_2) / 2$, where σ_1 and σ_2 are the diameters of the hard cores of the particles, and the energy scale is set to 1 kT. For the Gaussian interactions between polymer blobs, the potential takes the functional form $U_{gauss}(r) = -A \exp(-Br^2)$ for $r < r_{cut}$, where the length-scale parameter is $B = 0.8 / [(R_{g,1} + R_{g,2}) / 2]^2$ and $R_{g,1}$ and $R_{g,2}$ are the radii of gyration of the blobs in dilute solution[S2]. The cutoff distance is set to $r_{cut} = 1.5\ (R_{g,1} + R_{g,2})$. The interaction-strength parameter, A, is chosen to be a constant value of 8 kT for all interactions among N-terminal and C-terminal BRD4 blobs; however, because of the differing σ and R_g values of these species, the resulting well depths for the summed WCA and Gaussian potentials differ as well ($U_{min,NN}/kT = -0.75$, $U_{min,CN}/kT = -1.2$, $U_{min,CC}/kT = -1.9$). These choices lead to the reproduction of the experimentally determined phase diagram as demonstrated in the main text. The interaction-strength parameter, A, between acetylated histone tails and the N-terminal BRD4 blob is varied to represent either weakly attractive interactions with $A = -25\ kT$ (resulting in a second virial coefficient for the blob-blob interactions of $b_2 = B_2/V_{endog} \sim -0.5$, where $V_{endog} = (4/3\ \pi)\ \langle R_{g,\ endog} \rangle^3$) or strongly attractive interactions with $A = -50\ kT$ (resulting in $b_2 \sim -25$).

Table S1. Force-field parameters.

Parameter	Symbol	Value	Description
Occupied-volume diameter of the C-terminal blob (C)	σ_C	$1\ \sigma = 4.94$ nm	678 disordered amino acids (AAs)
Occupied-volume diameter of the N-terminal blob (N)	σ_N	$1.32\ \sigma$	361 disordered AAs + folded domains
Occupied-volume diameter of the Ferritin core (F)	σ_F	$2.43\ \sigma$	From Bracha et al. 2018[S3]
Occupied-volume diameter of the nucleosome (H)	σ_H	$2.02\ \sigma$	From Cutter and Hayes 2015[S4]
Occupied-volume diameter of the histone-tail blob (T)	σ_T	$0.354\ \sigma$	30 disordered AAs
Radius of gyration of the C-terminal blob	$R_{g,C}$	$0.818\ \sigma$	Ideal polymer model for 678 disordered AAs
Radius of gyration of the N-terminal blob	$R_{g,N}$	$0.847\ \sigma$	Ideal polymer model for 361 disordered AAs and folded domains
Radius of gyration of the histone-tail blob	$R_{g,T}$	$0.171\ \sigma$	Ideal polymer model for 30 disordered AAs
FENE bond spring constant for N-C bonds	$K_{FENE,NC}$	$2.886\ kT/\sigma^2$	Ideal polymer model
FENE bond spring constant for F-C bonds	$K_{FENE,FC}$	$1.937\ kT/\sigma^2$	Ideal polymer model

FENE bond spring constant for H-H bonds	$K_{FENE,HH}$	5.0 kT/σ^2	Ideal polymer model
FENE bond spring constant for T-H bonds	$K_{FENE,TH}$	5.704 kT/σ^2	Ideal polymer model

Table S2. Simulation parameters for nucleation simulations.

**Parameter**	**Symbol**	**Value**	**Description**
Simulation temperature	kT	1 ε	Energy scale
Timestep for the molecular dynamics integration	dt	0.005 τ	
Langevin damping parameter	$\tau_{langevin}$	10 τ	>> velocity autocorrelation decorrelation time
Number of Corelets in Corelet nucleation simulations	$N_{corelet}$	512	> 10 times the typical critical nucleus, N^*
Number of BRD4 molecules in endogenous nucleation simulations	N_{endog}	5832	> 40 N^*
Size of a stable cluster of endogenous BRD4 molecules	$N_{nucl,endog}$	600	> 4 N^*
Size of a stable cluster of corelet molecules	$N_{nucl,corelet}$	200	> 4 N^*
Size of a minimal registerable cluster of endogenous BRD4 molecules	$N_{reg,endog}$	~43400	D_{reg} ~ 360 nm<br>$\rho_{condense}$ ~ 1.776e-3 nm^{-3}
Size of a minimal registerable cluster of Corelet molecules	$N_{reg,corelet}$	~3070	D_{reg} ~ 360 nm<br>$\rho_{condense}$ ~ 1.256e-4 nm^{-3}

Supplementary Information References

S1. Weeks, J. D., Chandler, D. & Andersen, H. C. Role of repulsive forces in determining the equilibrium structure of simple liquids. *J. Chem. Phys.* **54**, 5237–5247 (1971).

S2. Louis, A. A., Bolhuis, P. G., Hansen, J. P. & Meijer, E. J. Can polymer coils Be modeled as 'Soft colloids'? *Phys. Rev. Lett.* **85**, 2522–2525 (2000).

S3. Bracha, D. *et al.* Mapping Local and Global Liquid Phase Behavior in Living Cells Using Photo-Oligomerizable Seeds. *Cell* **176**, 407 (2019).

S4. Cutter, A. R. & Hayes, J. J. A brief review of nucleosome structure. *FEBS Lett.* **589**, 2914–2922 (2015).

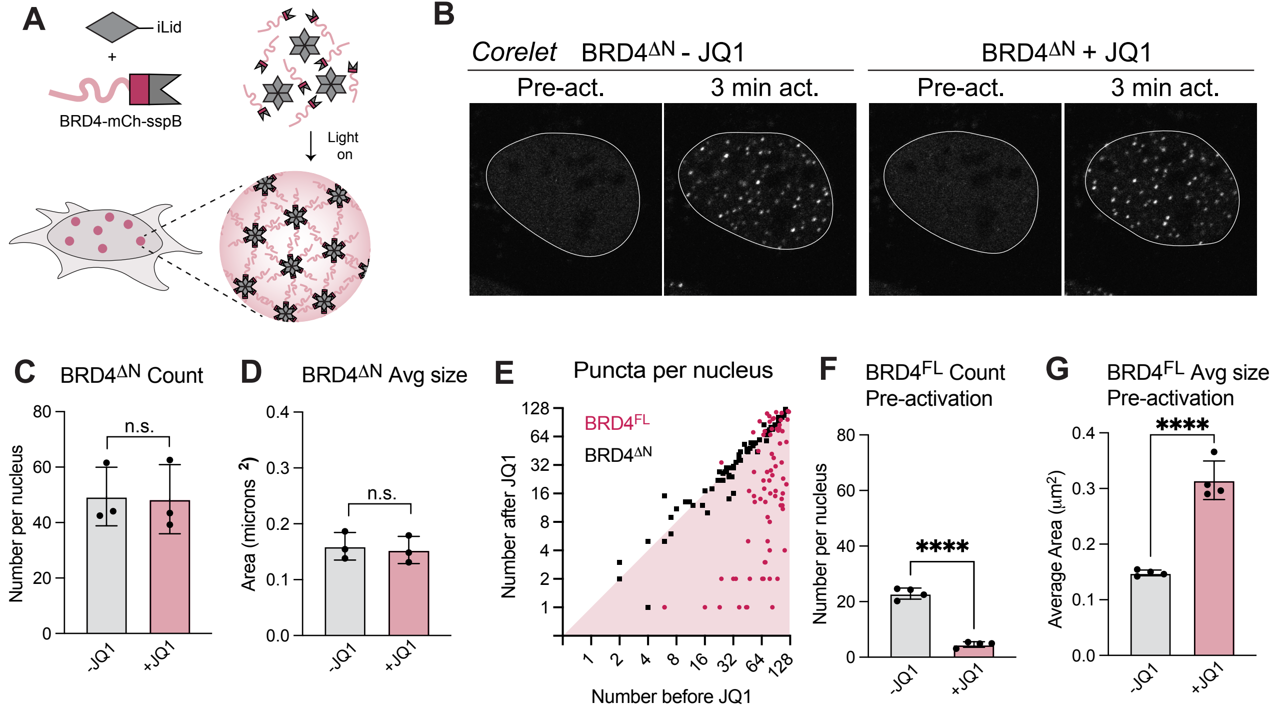
A
iLid
+
BRD4-mCh-sspB
Light on
B
Corelet
BRD4$^{\Delta N}$ - JQ1
BRD4$^{\Delta N}$ + JQ1
Pre-act.
3 min act.
Pre-act.
3 min act.
C
BRD4$^{\Delta N}$ Count
Number per nucleus
n.s.
-JQ1
+JQ1
D
BRD4$^{\Delta N}$ Avg size
Area (microns ^{2})
n.s.
-JQ1
+JQ1
E
Puncta per nucleus
BRD4^{FL}
BRD4$^{\Delta N}$
Number after JQ1
Number before JQ1
F
BRD4^{FL} Count
Pre-activation
Number per nucleus
****
-JQ1
+JQ1
G
BRD4^{FL} Avg size
Pre-activation
Average Area (μm^2)
****
-JQ1
+JQ1

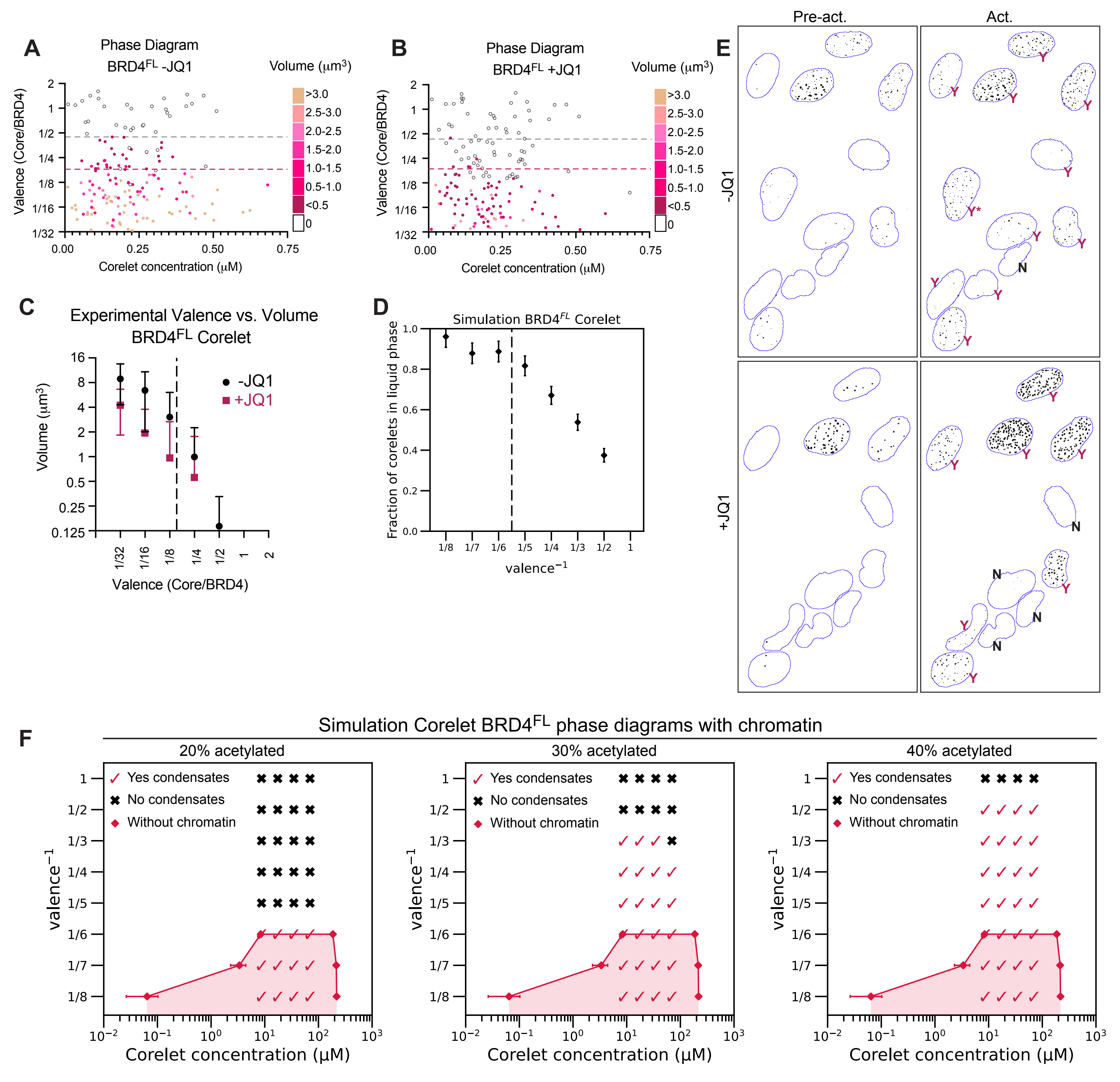
A
Phase Diagram
BRD4FL -JQ1
Volume (μm3)
>3.0
2.5-3.0
2.0-2.5
1.5-2.0
1.0-1.5
0.5-1.0
<0.5
0
Valence (Core/BRD4)
2
1
1/2
1/4
1/8
1/16
1/32
0.00
0.25
0.50
0.75
Corelet concentration (μM)
B
Phase Diagram
BRD4FL +JQ1
Volume (μm3)
Valence (Core/BRD4)
Corelet concentration (μM)
C
Experimental Valence vs. Volume
BRD4FL Corelet
Volume (μm3)
-JQ1
+JQ1
Valence (Core/BRD4)
D
Simulation BRD4FL Corelet
Fraction of corelets in liquid phase
valence−1
E
Pre-act.
Act.
-JQ1
+JQ1
Y
N
F
Simulation Corelet BRD4FL phase diagrams with chromatin
20% acetylated
30% acetylated
40% acetylated
Yes condensates
No condensates
Without chromatin
valence−1
Corelet concentration (μM)

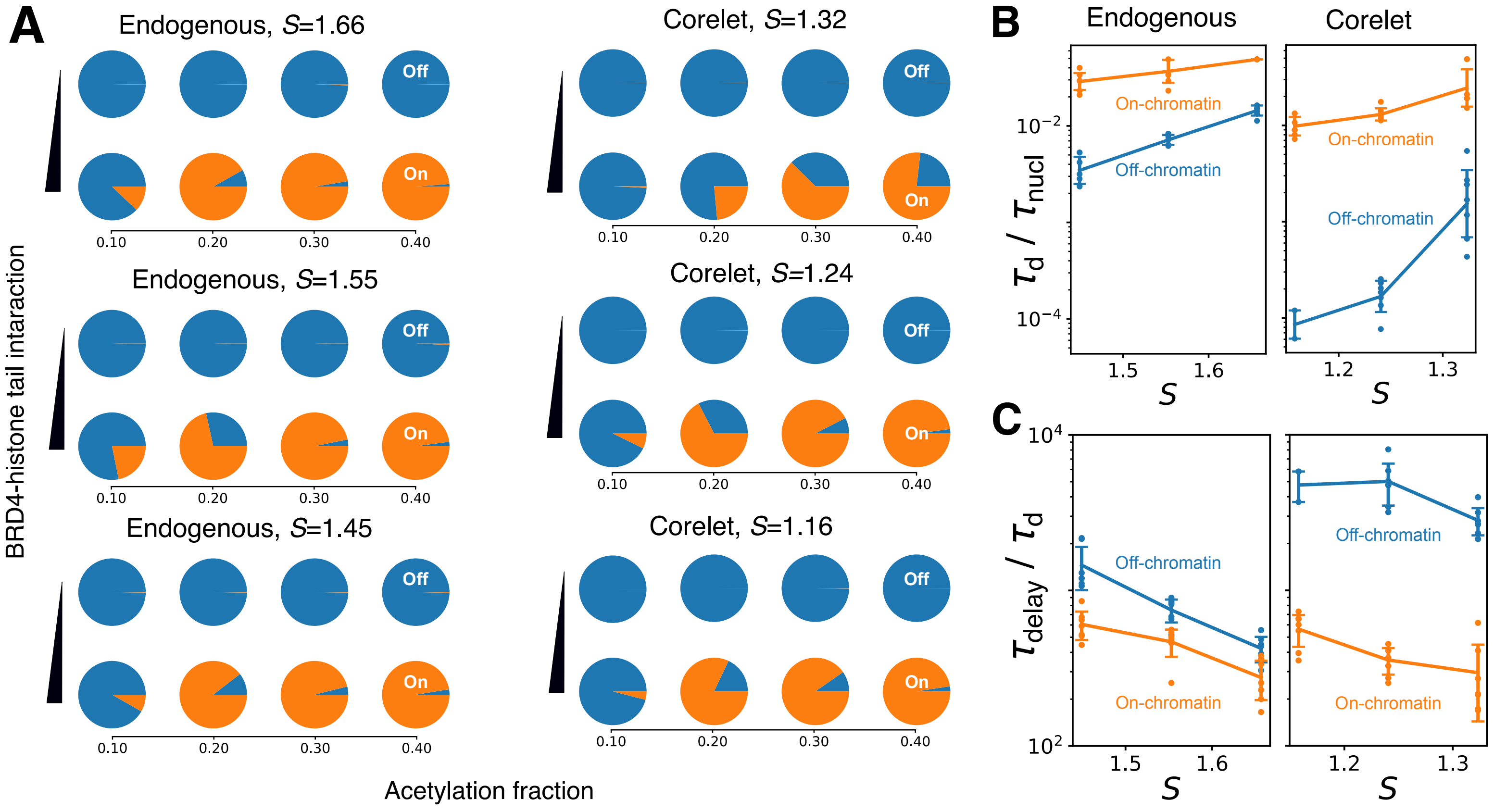

A
Endogenous, S=1.66
Endogenous, S=1.55
Endogenous, S=1.45
Corelet, S=1.32
Corelet, S=1.24
Corelet, S=1.16
Off
On
0.10
0.20
0.30
0.40
BRD4-histone tail intaraction
Acetylation fraction
B
Endogenous
Corelet
On-chromatin
Off-chromatin
τ_d / τ_{nucl}
10^{-2}
10^{-4}
1.5
1.6
1.2
1.3
S
C
τ_{delay} / τ_d
10^4
10^2

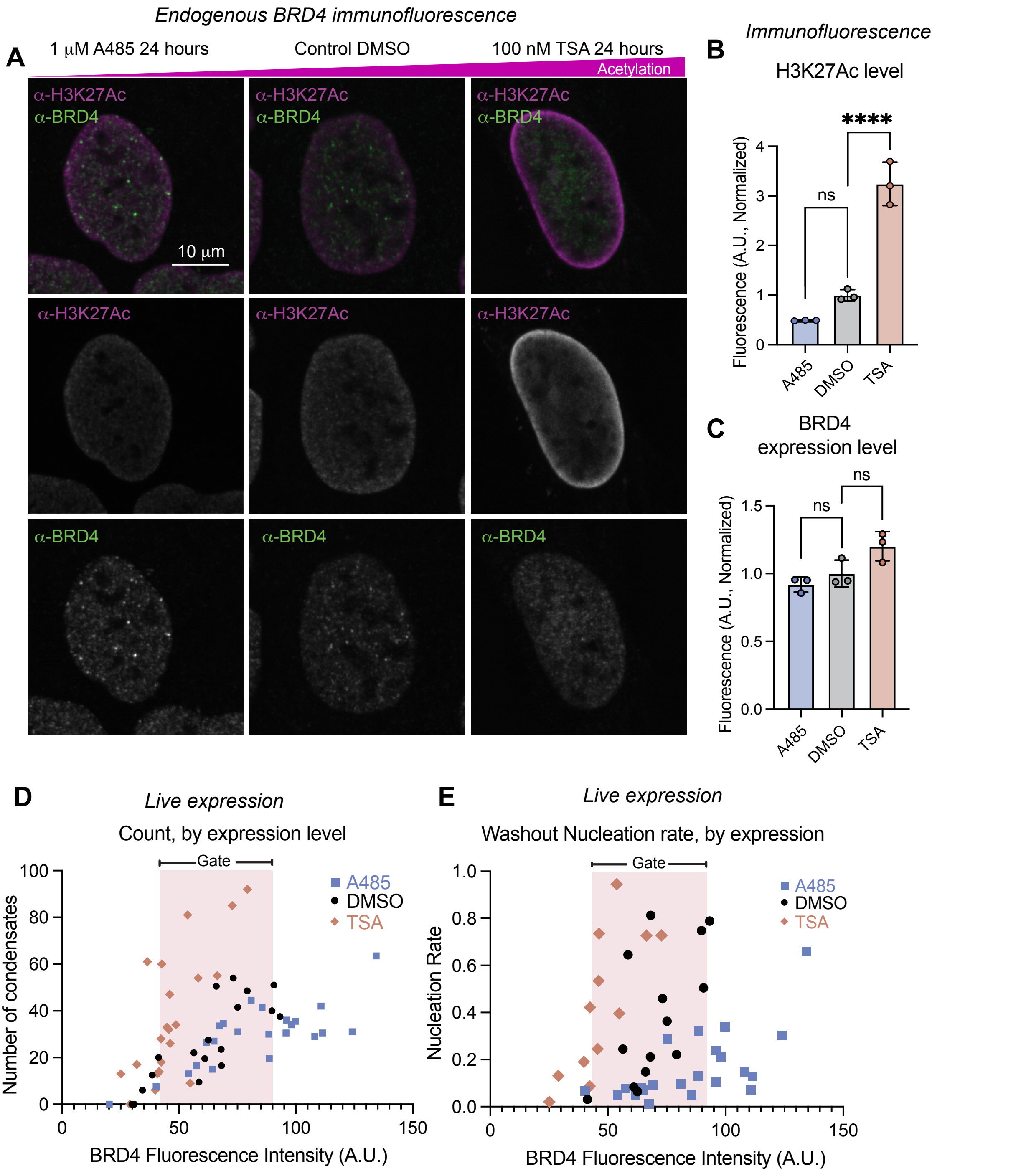

A
Endogenous BRD4 immunofluorescence
1 µM A485 24 hours
Control DMSO
100 nM TSA 24 hours
Acetylation
α-H3K27Ac
α-BRD4
10 µm
B
Immunofluorescence
H3K27Ac level
****
ns
Fluorescence (A.U., Normalized)
A485
DMSO
TSA
C
BRD4 expression level
ns
ns
D
Live expression
Count, by expression level
Gate
A485
DMSO
TSA
Number of condensates
BRD4 Fluorescence Intensity (A.U.)
E
Live expression
Washout Nucleation rate, by expression
Nucleation Rate
